# Assessing the evolution of primary healthcare organizations and their performance (2005-2010) in two regions of Québec province: Montréal and Montérégie

**DOI:** 10.1186/1471-2296-11-95

**Published:** 2010-12-01

**Authors:** Jean-Frédéric Levesque, Raynald Pineault, Sylvie Provost, Pierre Tousignant, Audrey Couture, Roxane Borgès Da Silva, Mylaine Breton

**Affiliations:** 1 Institut national de santé publique du Québec, Québec, Canada; 2 Centre de recherche du Centre hospitalier de l'Université de Montréal, Québec, Canada; 3 Direction de santé publique de l'Agence de la santé et des services sociaux de Montréal, Québec, Canada

## Abstract

**Background:**

The Canadian healthcare system is currently experiencing important organizational transformations through the reform of primary healthcare (PHC). These reforms vary in scope but share a common feature of proposing the transformation of PHC organizations by implementing new models of PHC organization. These models vary in their performance with respect to client affiliation, utilization of services, experience of care and perceived outcomes of care.

**Objectives:**

In early 2005 we conducted a study in the two most populous regions of Quebec province (Montreal and Montérégie) which assessed the association between prevailing models of primary healthcare (PHC) and population-level experience of care. The **goal **of the present research project is to track the *evolution *of PHC organizational models and their relative performance through the reform process (from 2005 until 2010) and to assess factors at the organizational and contextual levels that are associated with the transformation of PHC organizations and their performance.

**Methods/Design:**

This study will consist of three interrelated surveys, hierarchically nested. The first survey is a population-based survey of randomly-selected adults from two populous regions in the province of Quebec. This survey will assess the current affiliation of people with PHC organizations, their level of utilization of healthcare services, attributes of their experience of care, reception of preventive and curative services and perception of unmet needs for care. The second survey is an organizational survey of PHC organizations assessing aspects related to their vision, organizational structure, level of resources, and clinical practice characteristics. This information will serve to develop a taxonomy of organizations using a mixed methods approach of factorial analysis and principal component analysis. The third survey is an assessment of the organizational context in which PHC organizations are evolving. The five year prospective period will serve as a natural experiment to assess contextual and organizational factors (in 2005) associated with migration of PHC organizational models into new forms or models (in 2010) and assess the impact of this evolution on the performance of PHC.

**Discussion:**

The results of this study will shed light on changes brought about in the organization of PHC and on factors associated with these changes.

## Background

In early 2005 we conducted a study in the two most populous regions of Québec province (Montréal and Montérégie) which examined the association between prevailing models of primary healthcare (PHC) and population-level experience of care [1]. This study followed the launching of two reform policy initiatives by the Québec's Ministry of Health and Social Services: the creation of Family Medicine Groups (FMG) and the establishment of Local Services Networks (Local Networks) under the governance of Health and Social Services Centres [2]. FMGs were established to increase accessibility and continuity of care while Health and Social Services Centres (Local Centres) aimed at better coordinating and integrating services by creating territorially-defined Local Networks. Although these policies were respectively proposed in 2002 and 2004, implementation was only begun, for the most part, in 2005, coinciding with the conduction of the aforementioned study.

Four years later both reforms are well-established, and the question arises of how PHC models have evolved, what factors have promoted the evolution of PHC organizations, and how this evolution has translated into measurable effects at the population level. The decision-makers of the two regions have approached our research team to explore these questions. The study we conducted at the early phase of implementation of these reforms will provide us with a reference point for assessing the evolution of PHC organizations over a five year period. The study's goal is to assess the evolution of PHC organizations through the reform, identify factors associated with this evolution, and evaluate its association with the performance of PHC organizations and Local Networks. The knowledge generated by this study will help to further PHC reorganization efforts in various jurisdictions by better understanding factors that can promote organizational change and by better understanding the impact of this change on population-level experience of care.

Our project team includes researchers and decision-makers engaged in the co-production of relevant information in order to guide PHC reforms and optimize PHC service provision. By providing sound evidence for decision-makers and clinicians regarding factors related to the transformation of PHC organizations, we aim at supporting the implementation of PHC reform efforts and thus improve the performance of the healthcare system in addressing healthcare needs of Canadians.

### The current reform of PHC organization in Québec

Health and Social Services Centres (Local Centres) have been created by law [3], merging acute care hospitals, long-term care hospitals and Local Community Services Centres (CLSC) on a geographical basis. Their main objective is to lead to the implementation of Local Networks and to increase collaboration among PHC organizations through the creation of these networks [4]. The Local Networks are composed not only of the facilities merged under Local Centres but also of all other health and social services providers, including privately owned medical clinics. There are 95 Local Centres and Networks in Québec, 12 in Montréal and 11 in Montérégie. Local Centres and Networks vary in composition since some have acute care hospitals while others don't. In addition, Local Centres benefit from a large autonomy in the planning and organization of services and activities.

The FMG policy consists mostly in developing a contractual agreement between PHC clinics and the provincial government. PHC organizations receive complementary funding in exchange of complying with certain organizational requirements identified in the FMG policy (e.g. extended opening hours). In addition, each FMG has a contractual agreement with Local Centres that enables them to benefit from the presence of a nurse. A FMG consists of 6 to 10 physicians who work together with nurses to provide services for registered members of the group, on a non-geographical basis (usually around 10,000 to 20,000 people per FMG). A FMG provides services both by appointment and on a walk-in basis. It aims at being accessible 24 hours a day, 7 days a week, through opening hours that extend into the evening (until 9:00 p.m.) and weekends (at least 4 hours), and through a regional on-call system (Info Health line) for vulnerable patients when the clinic is closed. The target established at the start of the reform was to implement 300 FMGs in the province. As of March 2009, there were 181 accredited FMGs in Québec, 42 in Montréal and 55 in Montérégie.

A complementary model of organization currently being implemented in the regions under study is the Network Clinic. These clinical settings are more specifically targeted to ongoing and integrated management of clients, particularly those considered "vulnerable", and to provide access to basic technical support, such as radiology, blood tests, and specialists [5]. Their creation was initiated by the Montréal Regional Health Agency as a complement to FMGs, in response to requests by the regional medical association. A clinic can concurrently have the status of FMG and Network Clinic, thus benefiting from two sources of funding. As of March 2009, there were 36 Network Clinics in Montréal, among which twelve had both FMG and Network Clinic status.

### A recently completed research project

We recently completed the research project Accessibility and Continuity of Care: A Study of PHC in Québec which was conducted in two regions in the province--Montréal and Montérégie [1,6]. It looked at organizational models of primary healthcare and their influence on accessibility and use of health services by the population, as well as the experience of users of these services. The main objective of the study was to identify organizational models of PHC that are best adapted and most likely to meet the population's needs and expectations. The research included three components: 1) a survey of the population designed to measure utilisation of health services as well as users' perception of the accessibility, continuity, comprehensiveness, responsiveness and perceived results of services received [7]; 2) a study of PHC clinics that aimed to describe the PHC organization models in the regions studied [8]; 3) a contextual analysis that sought to describe Local Networks [9]. We identified five models of PHC organizations. Four were professional models (one was a single-provider model, one was a contact model (walk-in clinics), and two models were coordination models, one being integrated and the other non-integrated in the overall healthcare system), while one was a community-oriented model. Overall, the integrated coordination and single provider models were associated with better patient experience of care, followed by the community-oriented model. The contact professional model was associated with the worst experience of care across all measures [1].

### What does the literature tell us about PHC organizations?

Recent studies have focused on models of care, or ways to organize clinical services, that promote more accessible, coordinated, patient-centered care with emphasis on health promotion and disease prevention [10,11]. Models of care such as the medical home and the chronic care models, among the most often cited, have shown a great potential for achieving such results [11-15]. However, researchers have paid much less attention to the structure and processes developed at the organizational level, in which these models of care can be implemented and which require certain organizational conditions for their successful implementation [16].

Several organizational attributes have been associated with a better performance of PHC organizations [17]. For example, physician payment modalities have a determining effect on their practice. Fee-for service is associated with greater productivity but less continuity of care when contrasted with per capita prepayment which encourages more continuity and prevention [18,19]. Although it is possible to identify the effect of individual attributes of organizations on various process or outcome indicators, it remains more difficult to understand how these attributes relate to each other in actual organizations and systems. However, studies that focused on comparisons between different types of PHC organizations or systems (e.g. Kaiser or Veterans Administration) have provided enlightening results [20,21]. Although differences between types of organizations could be due to specific organizational attributes, understanding the effect of various organizational characteristics in a systemic perspective remains a challenge [22]. Hence, there is a need for a more holistic view in the study of healthcare organizations and systems.

The configurational approach, which views an organization as a whole rather than a set of independent attributes, is instructive in this regard [23,24]. This view seems to best meet the representation held by decision-makers of what an organization really is [25]. "In essence, a configurational approach suggests that organizations are best understood as clusters of interconnected structures and practices, rather than as modular or loosely coupled entities whose components can be understood in isolation" [26]. Configurations are "represented in typologies developed conceptually or captured in taxonomies derived empirically" [23]. Taxonomies are generally derived from cluster-analytic methods, thus forcing similar organizations to form homogeneous groups [26-29]. A complementary measure is a deviation score [30]. In this case, the researcher defines an ideal-type of attributes based on theoretical considerations and then calculates a score of conformity to this ideal-type, based on empirical observations [26].

One way to conceptualize various organizational models derived from the configurational approach is to consider them as a system for organized action defined by four sets of attributes: vision, resources, structure and practices [31]. As it applies to PHC organizations, vision corresponds to the values and representations shared by the actors [1,16]. Structure refers to the interaction and regulation among actors, such as interprofessional collaboration, and governance. Resources are defined by the type and level of various resources (human and material) and their arrangement. Finally, practices comprise mechanisms for offering services, developing multidisciplinarity and ensuring follow-up of patients.

This approach has been used in our previous work. In a recent policy synthesis, we derived a taxonomy of four models: two professional and two community models [16]. Following the same methodological approach, but using data on PHC organizations in two regions, we derived another taxonomy that is very consistent with the policy synthesis. We found only one community model, but four professional ones: the single provider, the contact, the coordination and the coordination integrated [1]. In order to contrast models from a normative standpoint, we also constructed an index of conformity to an ideal-type, based on the literature on group practice and on the various policy documents on new emerging forms of PHC organizations (such as the FMG).

Not only do these models or archetypes provide an holistic view of an organization, compared to other forms of organizations derived from the same taxonomy, but they also permit the assessment of change over time, when an organization passes from one archetype to another [23,25,30]. Comparing archetypes or models specific organizations belong to at different points in time is thus a sensitive measure of organizational change.

### What does the literature tell us about factors associated with PHC organizational change?

Institutional theory of organization has become widely used to explain organizational change [32-34]. According to this theory, the environment exerts a determining influence on organizations that tend to take a similar form within an organizational field (the sharing of common norms and values) leading to a certain degree of homogeneity called isomorphism [32,35,36]. In the public sector, geographically defined territories such as Local Networks can exert such an influence [37,38].

Environmental pressures exerted on organizations are of three types: coercive, normative and mimetic [36]. Coercive pressures refer to laws, regulations and state policies. As Scott [38] points out, the state has the definitive ability to apply these kinds of pressures either by law or by introducing strong incentives in financing publicly-supported organizations. The two measures introduced by the Québec Government to create FMGs and Local Centres are essentially of this kind. Normative pressures are very prevalent in an environment of professional organizations such as the healthcare system. They refer to values and norms held by professional associations that tend to permeate organizational boundaries [33,39,40]. Hence, local professional associations and leaders have normative influences on PHC organizations through their links with professionals in these organizations [38,39]. Finally, mimetic pressures stem from organizations considered as examples by others that tend to imitate them. FMGs and Network Clinics can be seen by other clinics as model PHC organizations, thus generating mimetic pressures on these clinics.

Although organizations within an organizational field tend to converge to some form of isomorphism in response to these pressures, they do not react exactly in the same manner [38]. There are intrinsic characteristics of organizations mainly related to dominant values held by their professionals and the role played by influential actors that make them more or less sensitive and receptive to these pressures [38]. For instance, clinics that already collaborate with other clinics may have a higher propensity to respond to mimetic or normative pressures [38].

These three types of pressure do not necessarily act in the same direction and they can even neutralize each other's influence. This was the case in the implementation of CLSCs (Local Community Services Centres) in Québec. The Government policy aimed to establish a public health and social services organization (coercive pressure) was opposed by professional medical associations which encouraged their members not to practice in CLSCs (normative pressure) and reactively developed a network of privately owned group practice clinics (mimetic pressure) [4,41]. The opposition and reaction of the medical organized medicine to the CLSC project was a major obstacle in making CLSC the point of entry into the system. This illustrates the point that in order to yield maximum organizational change these pressures need to align in the same direction.

### What does the literature tell us about the effects of PHC organizations in the context of reforms?

The contribution of PHC in achieving health objectives has been largely documented [42,43]. Systems based upon well-organized PHC are better performing in many aspects, namely experience of care (continuity, accessibility, comprehensiveness, responsiveness) [42,44]. They also report a more appropriate use of services, as reflected by a lower use of hospital and emergency care [45].

Reforms of PHC organizations and local organization of healthcare services have been the subject of various evaluative studies in Canada [46]. Studies in Québec, Ontario, Manitoba and British Columbia have highlighted the positive impact of new forms of PHC organizations integrating desirable attributes of experience of care [7,41,47-52]. Studies have focused on understanding the process of organizational changes using a case study approach [16,53], linking experience of care and use of services provided by a limited number of organizations [48,54], using administrative data files or population surveys. None of these studies have nominally linked services users with their regular source of care [55-57]. Overall, these studies have highlighted some benefits of emerging models of PHC in various provinces, with community-oriented models and those promoting coordination of care showing the best results regarding the experience of care of patients and regarding professional collaboration and satisfaction.

### The gap in knowledge and need for evaluating PHC reforms

Ongoing or recently completed studies in Québec focus on various aspects of organizational performance [1,48,53]. One study explored factors associated with the implementation of FMGs [53]. A multiple case study approach found a positive association between nurse-physician collaboration and experience of care [58]. An ongoing study using a cross-sectional design is looking at the relationship between types of PHC organizations and experience and quality of care [59]. A study currently underway adopts a longitudinal perspective to look at the implementation of Local Centres and the impact on utilization and experience of care [60].

To our knowledge, no studies have assessed the evolution of PHC organizational models, identifying factors that can explain changes, and their impact on population-level indicators. In addition, we did not find studies that have assessed the impact of PHC reforms on the level of inter-organizational collaboration. Our study includes all PHC organizations in two large regions, a sample of the population with representativeness at the Local Network level and nominal linkage with the regular source of care. This evaluation of the evolution of models of PHC and of its population-level impact is required to guide the continuation and completion of the PHC reform and assess the improvement in capacity to respond to needs and expectations of populations. Such knowledge is crucial given the difficulties of reforming PHC in pluralistic contexts, such as Canada, and the relatively high costs that such reform demands. Decision-makers need to understand what promotes organizational change and how change and its benefits may be sustained.

### Conceptual framework

**Figure 1:**
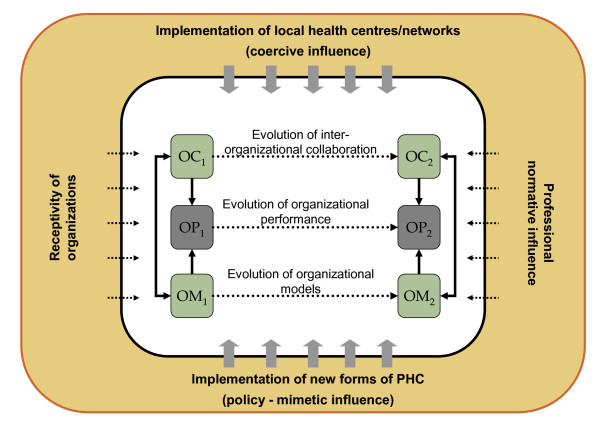
**Conceptual framework**.

Our conceptual framework is presented in figure 1. According to this framework, organizational models (OM) of PHC and the inter-organizational collaboration (OC) between PHC organizations influence the organizational performance (OP) of PHC systems. In addition, certain factors have an impact on the evolution of PHC organizational models and on inter-organizational collaboration through a period of transformation (Time 1 and 2). These factors relate both to the policies established by the Governments and to more implicit organizational environments.

The implementation of Local Centres and Networks is seen as exerting a coercive influence on the evolution of PHC organizations. We expect the integrating influence of Local Centres will increase networking as expressed by inter-organizational collaboration among all organizations within the territory. Specific interventions or regulations can in fact influence the ways PHC settings organize various aspects of care. Examples of such interventions can include the funding of specific initiatives by local health authorities, development of specific organizational projects under the impetus of coordinating bodies or modification of relationships between organizations because of restructuring services at various levels of Local Centres and Networks. The introduction of a new organization policy has a direct effect on the implementation of emerging forms of PHC such as FMGs through explicit policies aimed at promoting change in the way care is organized. The implementation of new forms of organizations can also have a mimetic influence on the other forms of PHC organizations and the inter-organizational collaborations in place.

In addition to these contextual influences, some characteristics and attributes of PHC organizations make them proactive or more receptive towards change. These attributes can be related to the presence of a designated team leader, or their organizational culture (e.g. concordance between dominant organizational values and current proposals of reform). Professional influence relates to the presence of leaders and professional organizations that apply pressure on PHC organizations towards accepting or opposing changes. These influences include elements such as the official position of medical representatives regarding specific policies or the presence of a local champion promoting a specific model of PHC organization.

These changes are expected to translate into an increased organizational performance at two levels: first, at the level of the clientele of these organizations and second at the level of the populations of each Local Network. We use performance here in a very broad sense to include various indicators of effects of PHC organizations [61]. We expect that change towards new forms of organizations at the level of Local Networks will be associated with improved population coverage (e.g. affiliation with regular sources of care and unmet needs for care), process of care (utilisation of services and patients' experience of care such as accessibility, continuity, comprehensiveness, responsiveness) and outcomes of care (e.g. perceived results of care, reception of preventive services, preventable hospitalizations and emergency room consultations) (see Additional file 1 for details of measures).

### Study objectives

The goal of this research project is to understand the evolution of PHC organizational models and their relative performance through the process of PHC reform, and assess factors, at the organizational and contextual levels, associated with the transformation of PHC organizations and their performance. More specifically, the objectives are:

1. to assess the magnitude and direction of organizational change and migration among models of PHC, between 2005 and 2010, at the PHC organization and Local Network levels as expressed by: 1) the prevalence and local configuration of PHC organizational models; 2) conformity of PHC organizations to a normatively defined ideal-type of organizational characteristics; and 3) the degree of collaboration between PHC organizations within and outside the Local Network;

2. to determine the association of these organizational changes of PHC with factors related to the implementation of Local Networks and policies aiming at promoting new forms of PHC organization, as well as factors related to the receptivity of PHC organizations and the influence of professional associations;

3. to examine the association between these organizational changes and various indicators of PHC performance (coverage, process and outcomes of care), both at the organizations' clientele and the Local Networks' population levels.

## Methods/Design

### Overall study design

**Figure 2:**
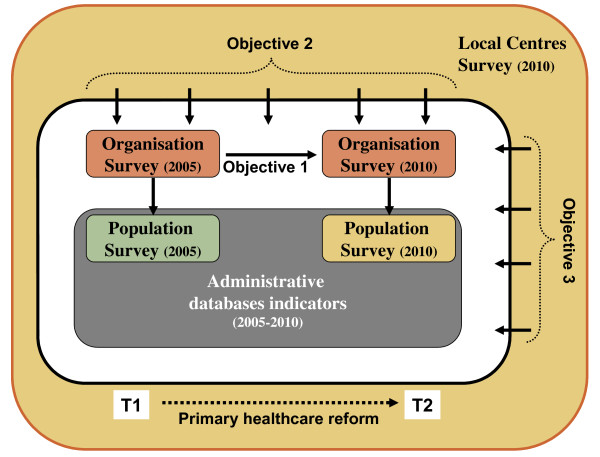
**Study design**.

This study employs a mix of cross-sectional and retrospective longitudinal design methods. It is also hierarchical in nature with nested levels of observation: individuals being affiliated to PHC organizations, which are located within specific Local Networks. This study will draw from four different sources of data to address the identified research questions. These four sources of data consist of: 1) individual-level data from a population survey of people's utilisation and experience of PHC; 2) individual-level data from administrative databases; 3) organizational-level data from a survey of PHC clinics; 4) contextual-level information from a survey of Local Centres (cf. figure 2).

Data collected during the period of PHC reform ranging from 2005 and 2010 will be used. The organizational and population-level data from 2005 will come from our previously conducted study of the impact of PHC organization models on experience of care of populations [1,6]. New organizational and population-level surveys will be conducted in 2010 as part of this research project to reassess organizational models and configurations as well as population-level coverage, processes and outcomes five years into the reform. Retrospective administrative data covering the reform period and a survey of Local Networks will complement these data sources. Additional file 2 summarizes the research themes, data sources, measurement tools and methods.

### Sources of data

An organization survey questionnaire will be mailed to all 665 PHC organizations in the selected regions, in 2010. We will use a previously developed survey of organizations (Additional file 3) focusing on their vision, material, financial and human resources, current organizational structures, and organizational practices supporting service delivery as well as inter-organizational collaboration [8]. Strong input from the research and decision-maker team members will help promote a high response rate from PHC organizations. A total of 473 organizations participated in the study conducted in 2005, for a response rate of 71% (66% in Montréal and 81% in Montérégie) [1]. The various types of private and public PHC organizations were well represented (solo, group, CLSC, family medicine units, and FMG) in that survey.

We will conduct, in 2010, a contextual appraisal of Local Networks (n = 23) using a survey tool developed in collaboration with another currently funded research team [62]. This tool will assess the Local Network's characteristics with regards to interventions aiming at promoting organizational change and inter-organizational collaboration at the PHC level. Key informants selected on a purposeful basis in each Local Centre will include a management level decision-maker, and a local representative of medical associations. This survey will be complemented with information coming from the organization survey pertaining to the clinics' perceptions about various aspects of their organizational context and the roles played by Local Centres in the reconfiguration of PHC (questions in Other Application Materials section) (see Additional file 4).

Concurrently with these two surveys, we will conduct a telephone population survey of randomly-selected community-dwelling individuals aged 18 and over in the 23 Local Networks of Montréal and Montérégie regions (400 respondents in each Local Network; total sample of 9200 respondents) using the random-digit dialling method. This survey of a representative sample of the population will enable us to measure people's affiliations with PHC organizations, utilization of healthcare services and unmet needs for care, selected attributes of people's experiences of care (accessibility, continuity, responsiveness, comprehensiveness), as well as perceived outcomes of care. We will use a previously developed questionnaire (Additional file 5) including validated indices of experience of care [7]. Based on our previous work, we can expect good rates of participation in the survey, with response rates of 63% in Montréal and 66% in Montérégie (Pineault et al., 2004; Pineault et al., 2009). In order to link persons with their associated organizational model of care, we will ask participants in the population survey to identify their usual source of care using a previously developed algorithm based on validated lists of PHC organizations in the two surveyed regions (this methodology has been validated in our previous survey).

To complement the information available through population surveys, we will use administrative databases comprising information regarding medical services (RAMQ), hospital-based services (Med-Echo), pharmaceutical prescriptions (Pharmacare), admission in long-term care facilities and death registry. The information gathered will cover the full population of the two regions and the complete span on time ranging from 2005 to 2010. The list of indicators is provided in Additional file 1.

#### Analytic theme 1: Assessing the magnitude of organizational change and collaboration (Objective 1)

The definition of "organization" used in this study refers to organizational entities that include one or several general practitioners offering general medical services. Therefore, private single-doctor offices are regarded as "organizations". Offices and clinics with more than one physician are also considered "organizations" whether or not physicians share a minimum number of resources (rooms, secretarial services or archives), and regardless of their degree of integration.

To assess the magnitude and direction of organizational change between 2005 and 2010 at the PHC organization and Local Network levels, we will use the organizational measurement tool developed as part of a previously funded project [8]. Using a hierarchical classification program applied in the previous project, we will construct an organizational taxonomy based on 2010 data and we will allocate all the organizations into models of this taxonomy through the classification component of this program [27,28]. This will provide us with a sensitive measure of organizational change. We will then assess in 2005 and 2010: 1) the prevalence and local configuration of PHC organizational models; 2) conformity of PHC organizations to a normatively defined ideal-type of organizational characteristics; and 3) the degree of collaboration between PHC organizations within and outside the Local Network.

The distribution of organizations on all variables of change will be compared in 2005 and 2010, globally for the two regions, and for each Local Network territory. To assess the migration of organizations from a model of organization to another between 2005 and 2010, two-level regression models with organizations nested within territories will be constructed, adjusting for 2005 results. The dependent variable corresponding to the taxonomy of the organizations will be dichotomous or multinomial, depending on the focus of analysis (single models vs multiple models comparisons). In addition, regression models will be developed to predict the change in conformity score and level of collaboration (continuous dependent variables) at the two times of the study. Two-level linear models (nj = 23; nk = 450) will be built for both categorical and continuous dependent variables.

The hierarchical models will be developed by the predetermined introduction of blocks of variables related to the three levels of analysis. Empty models will be developed to assess the level of variance comprised at each level of analysis. Intra-class correlations and proportion of variance explained at each steps of model building will be calculated to guide the selection of the most appropriate models. The modelling strategy will include fixed as well as random effect models. Bootstrapping methods could be employed to develop robust estimates of effect. Appropriate statistical packages will be employed to conduct descriptive and multilevel analyses (HLM; SAS; STATA).

#### Analytic theme 2: Identifying organizational and contextual factors associated with organizational change (Objective 2)

To determine the influence of factors associated with the implementation of Local Centres and new PHC forms, as well as receptivity of PHC organizations and the influence of professional associations, on the changes assessed in Analytic theme 1, we will draw on information from the organization questionnaire, as well as from the questionnaire addressed to Local Centres' key informants.

Local Network level information and organizational level covariates will be added to the two-level regression models described in Analytic theme 1, using these variables as predictors for change in PHC organization at the local level and in inter-organizational collaboration. As in Analytic theme 1, our analysis will comprise all PHC organizations (approximately 450) and all Local Networks of the two regions (23) in 2010, paired with organizations and Local Networks in 2005. Current knowledge about hierarchical modelling suggests that these sample sizes will provide sufficient statistical power to assess the association of factors with organizational changes [63-65]. The same model building strategy as in Analytic theme 1 will be employed.

#### Analytic theme 3: Assessing the impact of organizational change on the performance of PHC models (Objective 3)

To address objective 3, aiming at examining the association between these organizational changes and various indicators of PHC performance, we will use data from the organizational and population components of this study. From the population questionnaire, we will calculate indicators of affiliation with a primary care provider, indicators of utilisation of healthcare services and indices of PHC experience, as validated in our previous study. Using the administrative databases of the entire studied population, we will calculate indicators of utilisation and outcomes of care, such as hospitalisation for ambulatory-care sensitive conditions (see Additional file 1 for details on the indicators). These various indicators from the population survey and administrative databases will be used to contrast the level of performance of different models of PHC organizations in 2005 and 2010 as well as comparing performance at the Local Network level during this period. Hierarchical models will be constructed to identify the organizational factors associated with better results regarding these indices of performance of PHC at the two different times of the study, controlling for age, gender, economic status and morbidity, and for the nesting of individual observations in organizational settings and of organizations in Local Networks settings (three-level models). These models will include a time indicator (2005 vs 2010) as well as same- and cross-level interactions to test magnitude and correlates of change in performance of PHC. Particular attention will be given to the relationship between these indicators and sociodemographic and socioeconomic characteristics such as gender and vulnerability. The sample size will include more than 18,000 persons corresponding to the pooling of the two independent samples of population surveys in 2005 and 2010. The same model building strategy as in Analytic theme 1 will be employed.

### Power calculation

Power calculation always poses challenges in multilevel modelling. While it is well known that multilevel modelling is one of the most efficient statistical analysis when data structure involves nesting of data between levels, precise power calculation methods remain under development. However, some general rules guiding decisions regarding sampling and analyses in nested design exist. In this study, three levels of nesting are present. Power is influenced by the smallest sample size in the hierarchical structure. In this study, some analyses will use the 23 local health networks. However, most analyses will analyse the data taking the organisational level as the smallest sampling size. This level will include more than 300 observations with an average of 11,6 respondents per organisations. Despite variations in the number of respondents inside each organisations, this sample is equivalent to the suggested standards in the literature [63-65].

In addition, our design enables us to provide a power calculation based on our most demanding analysis, which is the multilevel analysis of theme 3, where patients are nested in Local Networks. To calculate the statistical power, we adopted the method of Snijders and Bosker [64], who proposed to divide the size of the sample by the design effect to obtain the size of the effective sample. Analyses can then be conducted as T-test differences for two independent samples with the size of the effective sample. Since in 2005 around 900 subjects were in the least frequent category of organizational model and the design effect was 1.48 (1.34 and 1.66 in each of the regions), the effective sample would be between 450 and 900 subjects for the least used organizational model. This allows us to detect a difference between 0.13 and 0.19 unit of standard deviation, with an α of 0.05 and a power of 80%. According to Cohen [66], this difference can be considered as a weak effect. As our calculation is based on comparisons involving the smallest numbers of subjects, our method of calculation remains conservative.

### Study limits and strengths

As with any study using respondents' perceptions (population survey, organizational survey), this study could suffer from perception bias and desirability bias, individuals being reluctant to be critical of their PHC clinic services and organizational respondents giving a biased portrait of their organization's characteristics. However, this bias should affect each type of organization in a similar way and be conservative. In addition, we will benefit from information coming from administrative databases and will be able to compare perceptual information with harder data collected through these databases.

Another limitation is that the information will come from a single province and will assess specific aspects of performance of PHC organizations. Other aspects such as economic productivity, technical quality of care or impact on health outcomes are not easily measured by population and organizational surveys. However, this survey will provide the first in-depth analysis of a PHC system, providing population coverage. In addition, our knowledge translation plan includes a national advisors meeting to discuss the applicability of our results to other Canadian contexts.

This study also benefits from specific strengths. First, the use of both taxonomic approaches and single characteristics assessments will enable the researchers to assess the impact of organizations in light of their complexity as well as identifying certain key characteristics that can have a specific impact on their performance. Furthermore, its longitudinal design and nominal link between users and their PHC organizations will enable the research team to assess the directionality of associations being measured, something missing from many cross-sectional surveys of PHC organizations performance. Finally, the explicit conceptual framework used in this study will enable the research team to test appropriate hypotheses with a clear explanatory framework to guide the co-creation of knowledge between decision-makers and researchers.

### Knowledge translation and exchange plan

Our knowledge translation and exchange (KTE) strategy is based on the conceptual formulation presented by Klein [67], who distinguishes three types of evidence: scientific, organization and political. Scientific evidence is produced by researchers. Organizational evidence concerns the feasibility of solutions emerging from scientific research. Finally, political evidence looks at the desirability of these solutions. Each type of evidence addresses different target audiences for KTE activities: the research community; the decision/policy makers, including politicians and pressure groups; and the general public. In addition to presentation during scientific meetings targeting exchange in knowledge among the scientific community, our KTE activities will target four specific audiences.

The first audience is the regional and Local Network levels, particularly the two regional health agencies, that participate in the coproduction and the financing of this research. The experience acquired in the preceding project and the links we have established with the decision-makers of the two agencies will facilitate our task in KTE activities. First, one of the decision-makers (D. Roy) is principal co-investigator of the project. Timeliness is an important condition for the use of research results by decision-makers [68]. Consequently, we will respond to invitations from the two agencies to present our preliminary findings, as soon as they become available. Our experience in the preceding project has taught us that decision-makers are mainly interested by descriptive data of the experience of care of their population and of their PHC organizations. We intend to repeat that strategy that will extend to Local Centres, as we will provide them with a picture of their territory.

A second audience is PHC clinicians. As in the preceding project, we will seize any opportunity to participate in the regional meeting of the Regional Department of Medicine of the two regions and to meet with the local medical associations. This proved to be very fruitful in the past, as we have established solid links with the medical leaders. In addition, at the end of the project, a feedback report will be sent to each participating clinic, showing the performance of the models of the taxonomy and to which model they belong. This feedback was greatly appreciated in the previous project and prepared the ground for future participation.

A third audience is the Ministry of Health and Social Services, that has expressed great interest in and support for our project. In May 2009, we are invited (JFL and RP) with Bill Hogg, to attend a one-day consultation meeting on the organization of PHC in Québec. We will meet on a regular basis with the persons responsible for the implementation of FMGs and evaluation of services, to keep them informed of our findings, as soon as they are produced.

A fourth audience is the general public. We can count on the support of our two institutions, the Institut national de santé publique du Québec and the Direction de santé publique (Public Health Department) de l'Agence de la santé et des services sociaux de Montréal, that have a great deal of expertise and experience in publicizing research to the general population. In addition, we have established strong links with the media in previous projects that will benefit this aspect of our KTE plan.

We expect that our KTE plan, by targeting these different audiences, will have a major impact. Knowledge translation activities will revolve around the collaboration with established groups (GETOS, GRGT, INSPQ) and use their established links and networks of knowledge exchange. Presentations to the various collaborating agencies will occur and scientific publications will be accompanied with policy-oriented timely documents. These documents will include descriptive reports related to the experience of care and organization of PHC at the local and regional levels, methodological reports related to the various components of the study and thematic reports focusing on policy-relevant subjects (e.g. unmet needs, PHC affiliation, access for vulnerable populations). The expertise of many of our team members on this front will ensure effective knowledge translation and exchange. Examples of reports recently produced by the research team are available in Other Application Materials section.

Finally, just before we produce the final report of this research, we will organize a national meeting on PHC, where we will share with researchers, decision/policy makers, and representatives of the public, the results of our research, along with those of other research teams, namely the Ottawa team, with which we have established collaborations. In preparation for this event, we will prepare a synthesis of findings produced by researchers in Ontario, Québec, and bC. This will be done following the methodology that we adopted in conducting a research collective and specifically in integrating the decision-makers' viewpoints in producing a synthesis [69-71].

## Competing interests

The authors declare that they have no competing interests.

## Authors' contributions

Understanding the evolution of PHC organizational models and their relative performance through the reform of PHC and assess the factors, at the organizational and contextual levels, associated with the transformation of PHC organizations and their performance, requires a diversity of skills and experience.

JFL, designated PI, will lead the project and be involved in all steps of the study, including knowledge translation and exchange (KTE). His experience as co-PI on project Accessibility and Continuity of Care: A Study of PHC in Québec (CHSRF-funded) will ensure continuity between T1 and T2 of the research. Through his experience in policy making and research in PHC, he has acquired unique expertise and skills to coordinate a research team of researchers and decision-makers. He read and approved the final manuscript. DAR, as Principal Decision-maker, will take part in the overall conduct of the study and strategic planning of research and KTE activities with the principal investigators. RP, co-PI, has led project Accessibility and Continuity of Care: A Study of PHC in Québec with Dr Levesque and will serve as senior investigator and mentor for the team. He will bring an important contribution to all components of the research, including KTE. He read and approved the final manuscript. PT is co-PI. As senior researcher and expert in disease surveillance, administrative database studies and population-based surveys, he will lead the databases indicator development component of this project (Theme 3). He read and approved the final manuscript.

The co-investigators will be involved more specifically in different components (Analytic themes) of the project. JLD holds a Research Chair from CIHR and CHSRF (cadre program) aiming at improving knowledge base and KTE with regards to the analysis of organizational change. He will contribute more specifically to Theme 2. PL has extensive experience in the analysis of healthcare policy and systems. His experience will be useful for the conduct of the organizational component of this research (Theme 1). SP has expertise in public-health surveillance, clinical preventive practices and surveys. She will be involved in the different components of this project and more specifically in Theme 3. She read and approved the final manuscript. MDB is the current holder of the Chaire Sadok-Besrour in Family Medicine. The expertise she brings to the team includes in-depth understanding of PHC organization and experience of care (Themes 1 and 3). DF will contribute to Theme 3 with her experience in outcome measurement, access to services, and use of administrative databases for health research. JH holds a Canada Research Chair on the population impact of primary healthcare organizations. Her extensive knowledge in measurement of patients' experiences with PHC will be contributive in Theme 3. DR was involved in the project Accessibility and Continuity of Care: A Study of PHC in Québec as co-PI with Dr Levesque and Pineault. Her extensive knowledge and research experience in qualitative research will be a great asset in Theme 2. JC has acquired a vast experience in integrated care for specific populations and will bring the viewpoint of nursing in the provision of PHC (Theme 1). MF is statistician. His expertise will be required in quantitative analysis throughout the project. MH was co-investigator and coordinator of the project Accessibility and Continuity of Care: A Study of PHC in Québec. She will ensure the link between T1 and T2 of the research, more specifically in Theme 1. AC will bring her assistance in the overall coordination of the project and will be involved more specifically in Theme 1. She read and approved the final manuscript. RBDS and MB are postdoctoral trainees who will be involved in organizational and contextual analyses respectively. They read and approved the final manuscript.

Co-decision-makers on the project will bring substantive support in KTE activities in their specific professional sphere: FG in the medical community; MD in public health; and JR in the linkage between physicians and decision-makers. LC is active in the implementation of new forms of PHC in Montréal region. His strategic position will facilitate KTE activities among top level decision-makers.

Finally, in order to achieve a bi-directional flow of information between investigators and decision-makers, we will establish an advisory committee composed of collaborators coming from clinical, management and policy fields. Regional and provincial bodies will also be part of this advisory group. This advisory committee will also benefit from the participation of recognized researchers in PHC from other Canadian provinces as well as national associations in order to broaden the scope of the study and ensure a larger transferability of the results. An innovative feature of our research program is the longitudinal nature of the program of research. We plan to complement the aforementioned areas of studies with inquiries suggested by the advisory committee throughout the program of research. This will enable the advisory committee to influence the ongoing processes of data collection and analyses. In return, this body will provide organizational insights into the findings.

## Pre-publication history

The pre-publication history for this paper can be accessed here:

http://www.biomedcentral.com/1471-2296/11/95/prepub

## Supplementary Material

Additional file 1 **List of indicators and measures**. This file lists indicators used for each of the three research themes along with their measures and sources. Click here for file

Additional file 2 **Research program grid**. This file specifies for each research theme the source of data, the measurement tools and the analytic procedures to be used. Click here for file

Additional file 3 **Organizational Questionnaire**. The organizational questionnaire contains all the questions completed by each PHC organization. It pertains to various aspects of these organizations such as vision, structure, resources and practices. The last section deals with the reorganization of PHC services. Click here for file

Additional file 4 **CSSS Questionnaire**. This file contains the questionnaire completed by key informants of Local Centres. It explores mainly the role played by Local Centres in developing interorganizational collaboration and networking as well as the support they gave to emerging forms of PHC organizations. Click here for file

Additional file 5 **Population Questionnaire**. This file contains the questionnaire used in the population survey. Questions relate mainly to experience of care, utilization and unmet needs. Click here for file
