# Supplementary material for: Assessing the evolution of primary healthcare organizations and their performance (2005-2010) in two regions of Québec province: Montréal and Montérégie
Source: BMC Fam Pract. 2010 Dec 1;11:95. doi: 10.1186/1471-2296-11-95 (PMC3014883; doi:10.1186/1471-2296-11-95)
Supplement: Additional file 1 — List of indicators and measures. This file lists indicators used for each of the three research themes along with their measures and sources. [file 1471-2296-11-95-S1.DOC]

Additional file 1: List of indicators and measures

| **Object** | **Indicators** | **Measures** | **Sources of data [[1]](#footnote-2)** |
| --- | --- | --- | --- |
| **Theme 1: Magnitude and direction of organizational change and collaboration / Comparison T1 – T2** | | | |
| **Organizational models of PHC** | Prevalence of the organizational models (regional and local levels) | - Model of the taxonomy corresponding to each clinic [[2]](#footnote-3) | Organization survey / T1-T2 |
| **Organizational conformity** | Conformity to an organizational ideal-type (regional and local levels) | - Ideal-type conformity scores of PHC organizations [[3]](#footnote-4) | Organization survey / T1-T2 |
|  | Individual characteristics of PHC organizations (tracers) (regional and local levels) | - Clinics where team work is valued - Clinics with nurses - Clinics with nurses with extended role - Clinics with 3 or more information technologies - Clinics where sharing of clinical activities is important - Clinics with a mix of consultation options (e.g., walk-in or by appointment and telephone consultations) - Clinics offering a broad range of services delivered | Organization survey / T1-T2 |
| **Collaboration**  **between organizations** | Collaboration between PHC organizations, within vs. outside Local Network territory: global and specific indices (regional and local levels) | - Clinics with clinical collaboration with other PHC organizations - Clinics where collaboration aims to: - coordinate services offer - share human and technological resources - refer or transfer patients - coordinate care to patients | Organization survey / T1-T2 / additional questions T2  Local Centres’ key informants survey |
|  | Collaboration between PHC organizations and hospitals, within vs. outside Local Network territory: global and specific indices (regional and local levels) | - Clinics with clinical collaboration with hospital - Collaboration between PHC organizations and hospitals regarding:   - access to clinical information   - access to diagnostic resources   - coordination of care |  |
|  | Perceived change in coordination between health care organizations  (regional and local levels) | Clinics perceiving changes since 2005 in mechanisms used to coordinate services to patients, as:   - - case discussion between professionals   - care protocols   - systematic follow-up   - case management | Organization survey / additional questions T2 |
|  | Regional and local coordination between health care organizations (regional and local levels) | - Clinics where physicians participate to regional committees - Clinics where physicians participate to a regional on-call network | Organization survey / T1-T2 |
| **Theme 2: Organizational and contextual factors associated with organizational change** | | | |
| **Implementation of Local Health Centres / Networks**  **(coercive influence)** | Interventions of the Local Centres in regards of:   - the implementation of the new organizational forms (FMG, NC) - the networking among all organizations within their territory   Perceived influence of the Local Centres | - Interventions of Local Centres to support implementation of FMG and NC - Interventions of Local Centres to support networking among organizations (concertation mechanisms, coordination structure, …) - Perceived influence of the Local Centres on the implementation of FMG and NC - Perceived influence of the Local Centres on the organizational networking | Local Centres’ key informants survey  Organization survey / additional questions T2 |
| **Implementation of new forms of PHC**  **(policy - mimetic influence)** | Saturation of Local Networks territories with new forms of PHC (regional and local levels) | - % of FMG in 2010 - % of NC in 2010 | Organization survey / additional questions T2 |
|  | Dominant organizational models within Local Networks territories | - % of each model within Local Networks territories in 2010 | Organization survey / additional questions T2 |
|  | Exemplarity | - Identification of clinics perceived as PHC organizations example to follow by other clinics (within Local Networks territories) - Identification of clinics and hospital perceived as leader in establishing inter-organizational collaborations | Organization survey / additional questions T2  Local Centres’ key informants survey |
| **Professional normative influence** | Interventions of professional medical associations in regards of:   - the implementation of the new organizational forms (FMG, NC) - the networking among all organizations within their territory   Perceived influence of the influence of professional medical associations | - Interventions of the FMOQ to support implementation of FMG and NC - Interventions of regional and local medical associations to support implementation of FMG and NC - Interventions of the regional and local medical associations to support networking among organizations (concertation mechanisms, coordination structure, …) - Perceived influence of the FMOQ on the implementation of FMG and NC - Perceived influence of the the regional and local medical associations on the implementation of FMG and NC - Perceived influence of the the regional and local medical associations on the organizational networking | Local Centres’ key informants survey  Organization survey / additional questions T2 |
| **Receptivity organizational factors** | Receptivity of the PHC organizations towards change (positive / negative) | - Presence of a designated team leader within the clinic - Concordance between the dominating organizational values and current proposals of reform - Motivation to become FMG or Network clinic | Organization survey / additional questions T2 |
| **Theme 3: Impact of organizational change on the performance of PHC models** | | | |
| **Experience of care** | Experience of care of the patient with regular source of care (regional, local and organizations’ clientele levels)  (Experience of care refers to the way individuals feel about or perceive or the care and services received; incorporates various attributes that can be evaluated by individuals, as accessibility, continuity, comprehensiveness, responsiveness or perceived outcomes of services. The regular source of care is the one reported by an individual as the place “you usually go to see a doctor for your general medical care, excluding specialised care". When respondents do not identify a usual place, the place where they went most frequently in the past two years is designated as the usual source of care.) | Global index of experience of care [[4]](#footnote-5) | Population survey and  Organization survey / T1-T2 |
|  |  | Geographical and organizational accessibility index |  |
|  |  | Economic accessibility index |  |
|  |  | Continuity of affiliation index |  |
|  |  | Informational continuity index |  |
|  |  | Responsiveness index |  |
|  |  | Comprehensiveness index |  |
|  |  | Perceived outcomes of care index |  |
| **Utilisation of health services** | Patient-reported utilisation of health services during last 2 years (regional, local and organizations’ clientele levels) | **Patient-reported utilisation of health services (%)**   - Health services non-users in the population - Only PHC users in the population - Population with at least one visit to the emergency room - Population with at least one hospitalisation   **Site of consultation (%)**   - Population having visited a physician in a CLSC - Population having visited a physician in a private clinic   **Reference (%)**   - Users having received a prescription for lab tests - Users refered to a specialist | Population survey and  Organization survey / T1-T2 |
|  | Utilisation of health services from administrative databases (regional and local levels) | **Utilisation of health services  (Population rates)**   - Visits to family physician - Visits to specialists - Emergency room visits - Hospital admissions   **Continuity and case management  (%)**   - Registration to FMG - Registration as vulnerable patient (RAMQ) - UPC (usual provider of care) index of continuity   **Preventive care practices**  **(Population rates)**   - Mammography within last 2 years (50 to 69 year-old women) - Smoking cessation counselling - STD counselling (18 to 25 year-old patients)   **Observance of chronic diseases care guidelines by PHC physicians (%)**   - prescription of ACE inhibitor for diabetic patients - prescription of β-blocker within 90 days after cardiac failure hospitalisation - prescription of ACE inhibitor within 90 days after cardiac failure hospitalisation   **Preventable emergency room visits (Population rates)**   - Emergency room visits for acute PHC sentinel conditions (otitis, cystitis, upper respiratory infections, conjonctivitis) - Emergency room visits for chronic PHC sentinel conditions (diabetes, COPD, asthma) - Emergency room visits within 1 to 3 months after hospitalisation for diabetes, cardiac failure, COPD, asthma   **Hospitalisations (Population rates)**   - Hospitalisation for ambulatory-care sensitive conditions | Administrative databases (hospitalisations, medical services, pharmaceutical services) |
| **Unmet needs for care** | Unmet needs for care during last 6 months (regional, local and organizations’ clientele levels)  (Unmet needs for care describes a situation where a person perceives a need to obtain health services but does not) | - Unmet needs for care - Problem at the origin of unmet needs for care - Consequences of unmet needs for care | Population survey and  Organization survey / T1-T2 |
| **Coverage of the population** | Affiliation with regular source of care by the organizational models of PHC | - % of health care users per organizational model of PHC - Ratio of % of users by model to the % of organizations   (through identification of the regular source of care and link with organizational model of PHC) | Population survey and  Organization survey / T1-T2 |
| **Response to vulnerable clienteles** | Coverage of vulnerable clienteles by the organizational models of PHC | - % of health care users with great vulnerability within each organizational model - Ratio of % of users with great vulnerability by model to the % of organizations   (Great vulnerability index constructed based on individuals’ accumulation of 3 conditions and over conditions of vulnerability: (1) health condition: serious; (2) level of education: low; (3) financial situation: insufficient income; (4) poverty: perception of being poor or very poor; (5) age: ≥ 65 years) | Population survey and  Organization survey / T1-T2 |

1. Survey questionnaires are attached. [↑](#footnote-ref-2)
2. For details about the construction of the taxonomy, see p.56-69 in **Accessibility and Continuity of Care: A Study of Primary Healthcare in Québec - Research Report** [↑](#footnote-ref-3)
3. For details about the construction of the ideal-type conformity score, see p. 74 in **Accessibility and Continuity of Care: A Study of Primary Healthcare in Québec**  [↑](#footnote-ref-4)
4. For details about the construction of the indices, see p. 51-56 in **Accessibility and Continuity of Care: A Study of Primary Healthcare in Québec - Research Report** [↑](#footnote-ref-5)
