# Supplementary material for: Assessing the evolution of primary healthcare organizations and their performance (2005-2010) in two regions of Québec province: Montréal and Montérégie
Source: BMC Fam Pract. 2010 Dec 1;11:95. doi: 10.1186/1471-2296-11-95 (PMC3014883; doi:10.1186/1471-2296-11-95)
Supplement: Additional file 2 — Research program grid. This file specifies for each research theme the source of data, the measurement tools and the analytic procedures to be used. [file 1471-2296-11-95-S2.DOC]

Additional file 2: Research program grid

| **Research themes** | **Data source** | **Measurement tools** | **Methods** |
| --- | --- | --- | --- |
| **Analytic theme 1**. To assess the magnitude and direction of organizational change between 2005 and 2010 at the PHC organization and Local Network levels | PHC organization survey (2005 and 2010) | Organizational questionnaire assessing aspects related to the vision, the infrastructure, the resources, the practices and collaboration of the PHC organizations | Development of a taxonomy of PHC organizations using a configurational approach (2010)  Development of indices of conformity to an organizational ideal-type  Comparison (2005-2010) of:   - prevalence and local configuration of PHC organizations - conformity of PHC organizations to desirable characteristics (tracers) and to an organizational ideal-type - degree of collaboration between PHC organizations within and outside the local network |
| **Analytic theme 2**. To determine the association of the organizational changes of PHC with factors related to the implementation of Local Networks and policies aiming at promoting new forms of PHC organization, as well as factors related to the receptivity of PHC organizations and the influence of professional associations | PHC organization survey (2005 and 2010)  Local Centres’ key informants survey (2010) | Idem as above for organizational component  Contextual questionnaire aimed at policy makers and healthcare managers in local health authorities and department of general medicine, assessing the actions done at the local level to influence PHC organizations and inter-organizational collaboration | Construction of multiple hierarchical regression predictive models of change in PHC organizational models and characteristics, including Local Network level covariates and organizational covariates |
| **Analytic theme 3**. To examine the association between the organizational changes and various indicators of PHC performance | Organization survey  Population survey of PHC coverage, and experience of primary healthcare (2005 and 2010) | Idem as above for organizational component  Population questionnaire assessing aspects of coverage (affiliation and unmet needs for care), process (utilisation and experience of PHC) and outcomes of care (perceived results) | Idem as above for organizational measures  Calculation of indices of PHC performance: affiliation, utilisation, unmet needs for care, indices of care experience, population-level outcomes  Comparison of performance at the two times of reform (2005 and 2010) and construction of multiple hierarchical regression models to identify the organizational factors associated with better results regarding indices of performance of PHC at two different times of the study, controlling for age, gender, economic status and morbidity, and for the nesting of individual observations in organizational settings and of organizations in Local Networks settings |
|  | Administrative databases (from 2005 to 2010) | Validated set of population-based health care utilisation indicators calculated at the Local Network level | Comparison of population-based health care utilisation indicators at the two times of reform (2005 and 2010) |
