# Supplementary material for: Assessing the evolution of primary healthcare organizations and their performance (2005-2010) in two regions of Québec province: Montréal and Montérégie
Source: BMC Fam Pract. 2010 Dec 1;11:95. doi: 10.1186/1471-2296-11-95 (PMC3014883; doi:10.1186/1471-2296-11-95)
Supplement: Additional file 3 — Organizational Questionnaire. The organizational questionnaire contains all the questions completed by each PHC organization. It pertains to various aspects of these organizations such as vision, structure, resources and practices. The last section deals with the reorganization of PHC services. [file 1471-2296-11-95-S3.DOC]

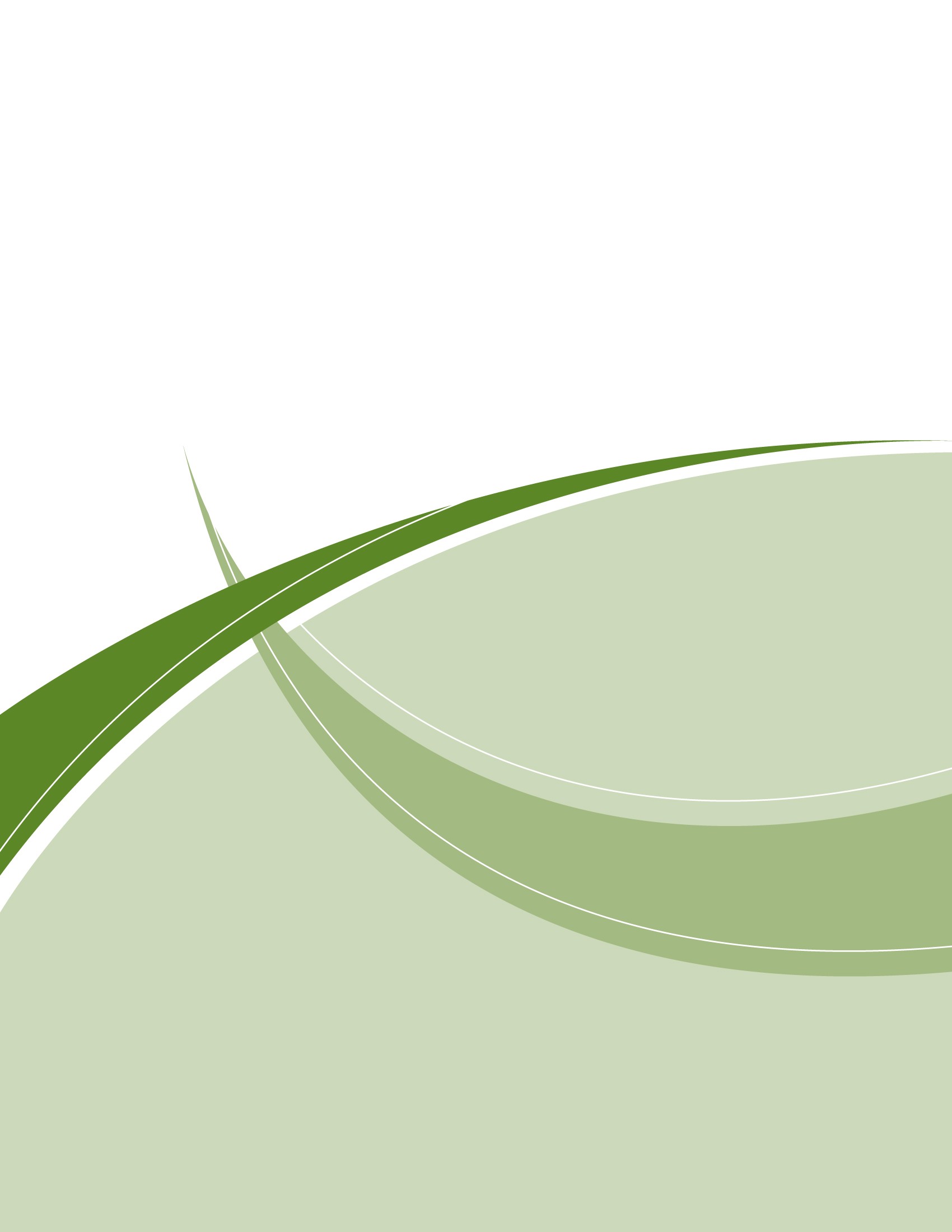

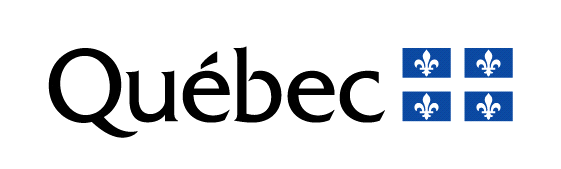
**Assessing the evolution of primary healthcare organizations**

**and their performance (2005-2010) in two regions of**

**Québec province: Montréal and Montérégie**

**Additional file 3:**

**Organizational Questionnaire**

July 21, 2010

Institut national de santé publique du Québec

Direction de santé publique, Agence de la santé et des services sociaux de Montréal


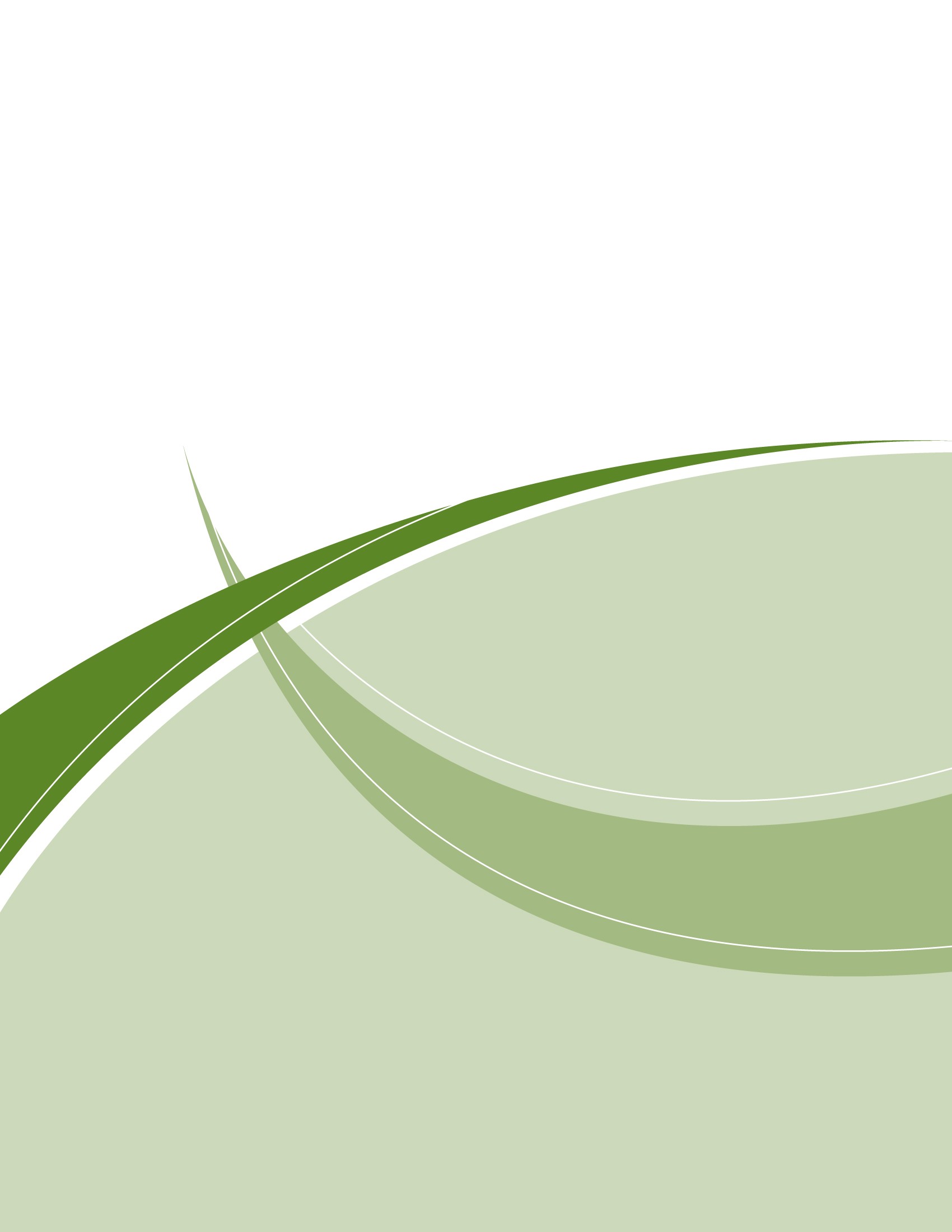


| **Instructions** |
| --- |

This is a questionnaire about the **organisation of primary healthcare services** offered in medical clinics, physician’s offices, polyclinics, local community services centres (*CLSC*), Family Medicine Groups (*FMG*)* and network clinics (*NC*)**. There is one questionnaire per clinic or civic address.

* Groupes de médecine de famille

** Cliniques-réseau

**WHO SHOULD ANSWER THIS QUESTIONNAIRE?**

The questionnaire must be filled in by the person who is most familiar with how the clinic is organised and operates; typically this is the physician-in-charge. For this reason, the questionnaire should not be completed by each physician at the clinic even when, in some clinics, physicians rarely work together.

**HOW SHOULD THE QUESTIONNAIRE BE COMPLETED?**

The consent form that is integrated to the questionnaire must be completed and signed.

Use of the expression ‘‘**your clinic**’’ refers to the primary healthcare medical team (general practitioners and nurses) to which you belong, or to yourself, if you are the only physician at the clinic. Answers should reflect as much as possible the views and practices of the entire primary healthcare medical team (general practitioners and nurses).

**Circle or check off ONE answer per question, unless otherwise indicated.**

**QUESTIONNAIRE COMPONENTS**

**Section A**: Resources and organisational structure

**Section B**: Services, practices and interorganisational collaboration

**Section C**: Vision/Mission and value system

**Section D**: Clinic location

**Section E**: Reorganisation of primary healthcare services

The questionnaire is also available on line. Please refer to the letter included with this questionnaire.

Si vous préférez recevoir ce questionnaire en français, veuillez contacter :

Olivier Juneau

Direction de santé publique de Montréal

Équipe Santé des populations et services de santé

Téléphone : 514 528-2400, poste : 3412

| **Section A: Resources and organisational structure** |
| --- |

1. **a) How many general practitioners, including those working part time, currently work at your clinic?** ______

**b) Indicate how many general practitioners in the following categories work at your clinic.**

- - - 1. Less than 10 hours a week? ______
      2. 10 to 25 hours a week? ______
      3. 26 to 40 hours a week? ______
      4. More than 40 hours a week? ______

**c) How many of these are in the following age categories?**

1. 34 and less? ______
2. 35 to 49? ______
3. 50 to 64? ______
4. 65 and more? ______

**d) How many of these are:**

1. women? ______
2. men? ______

**e) How many of these general practitioners have been working at your clinic for more than 5 years?** ______

- - - 1. **What is their main mode of remuneration for activities conducted at your clinic?** *Choose one answer only.*

1 Fee for service _2_ Sessional fees (vacation)_3_ Fixed honorarium _4_ Various modes of payment

1. **How many nurses currently work at your clinic?** ______
2. **At your clinic, is there anyone who…** Circle only one answer per statement.

|  | **No** | **Yes** | | | **Doesn’t apply because only 1 doctor in the clinic** |
| --- | --- | --- | --- | --- | --- |
|  |  | **A physician-in-charge or designated physician** | **The group of physicians collectively** | **Administrator/ manager** |  |
| 1. sets up on-call lists, schedules, vacation, etc.? | 4 | 1 | 2 | 3 | 99 |
| 1. organises meetings for case discussions? | 4 | 1 | 2 | 3 | 99 |
| 1. looks after recruitment of physicians and assigns practice privileges? | 4 | 1 | 2 | 3 | 99 |
| 1. ensures that the quality of medical acts is evaluated? | 4 | 1 | 2 | 3 | 99 |
| 1. organises continuing medical education activities? | 4 | 1 | 2 | 3 | 99 |
| 1. represents the clinic on committees? | 4 | 1 | 2 | 3 | 99 |
| 1. develops collective prescriptions/protocols for care? | 4 | 1 | 2 | 3 | 99 |

1. **Do any general practitioners at your clinic share …**

|  | **Yes** | **No** | **Doesn’t apply because only 1 doctor in the clinic** |
| --- | --- | --- | --- |
| 1. rooms (offices, examination rooms, waiting room)? | 1 | 2 | 99 |
| 1. operating costs for the clinic? | 1 | 2 | 99 |
| 1. support staff (secretary and receptionist)? | 1 | 2 | 99 |
| 1. an appointment management system? | 1 | 2 | 99 |
| 1. medical records system? | 1 | 2 | 99 |
| 1. pooled income? | 1 | 2 | 99 |

1. **Do any general practitioners at your clinic share…**

|  | **Yes** | **No** | **Doesn’t apply because only 1 doctor in the clinic** |
| --- | --- | --- | --- |
| 1. coverage of walk-in clinic periods? | 1 | 2 | 99 |
| 1. coverage of scheduled appointments periods? | 1 | 2 | 99 |
| 1. in-hospital care for clinic patients? | 1 | 2 | 99 |
| 1. patient follow-up? | 1 | 2 | 99 |
| 1. replacement for physician absent from the clinic? | 1 | 2 | 99 |

1. **To what extent do general practitioners at your clinic feel responsible for the health of the population in the neighbourhood, village or territory where your clinic is located?**

1 Highly _2_ Fairly _3_ Slightly _4_ Not at all

1. **To what extent do general practitioners at your clinic feel accountable for their professional activities to…**

|  | **Highly** | **Fairly** | **Slightly** | **Not at all** |  |
| --- | --- | --- | --- | --- | --- |
| 1. the RAMQ? | 1 | 2 | 3 | 4 |  |
| 1. the Collège des médecins? | 1 | 2 | 3 | 4 |
|  | **Highly** | **Fairly** | **Slightly** | **Not at all** | **Doesn’t apply because only 1 doctor in the clinic** |
| 1. colleagues at work? | 1 | 2 | 3 | 4 | 99 |
| 1. governance/clinic managers? | 1 | 2 | 3 | 4 | 99 |

- 1. **Does the funding for your clinic’s operating costs come from…**

1. fees charged to physicians or contributions by physicians?  1 Yes  2  No
2. private enterprises (companies, pharmacies, donations, foundation, etc.)? _1_ Yes  2  No
3. fees charged to patients (e.g. fees to open or manage files)? _1_ Yes  2  No
4. an institutional operating budget (CLSC, hospital)? _1_ Yes  2  No
5. infrastructure operating grant (FMG, Network-Clinic)?  1 Yes  2  No
6. **In your clinic, do you use …**
   1. computer software to manage appointments?  1 Yes  2  No
   2. Internet access (Web) for physicians? _1_ Yes  2  No
   3. access to the health and social services telecommunications network (RTSS)? _1_ Yes  2  No
   4. electronic medical records? _1_ Yes  2  No
   5. a Web-based appointment system for patients? _1_ Yes  2  No
   6. an electronic interface to diagnostic imaging laboratory services?  1 Yes  2  No
   7. an electronic system to transmit prescriptions to pharmacies? _1_ Yes  2  No
   8. computerized tools to aid medical decision-making (computerized alerts and recalls)? _1_ Yes  2  No
   9. computerized tools for continuing professional education? _1_ Yes  2  No
   10. practice clinical guidelines integrated with electronic medical records? _1_ Yes  2  No
   11. other **🢂 specify :** _1_ Yes  2  No
7. **Are the following services available in the building where your clinic is located?**
8. Blood samples   1 Yes  2  No
9. Radiology  1 Yes  2  No
10. Electrocardiography  1 Yes  2  No
11. Spirometry  1 Yes  2  No
12. Colonoscopy  1 Yes  2  No
13. Bone densitometry _1_ Yes  2  No
14. Magnetic resonance _1_ Yes  2  No
15. Ultrasound / Doppler _1_ Yes  2  No
16. Echocardiography  1 Yes  2  No
17. Computed tomography (CT)  1 Yes  2  No
18. Mammography  1 Yes  2  No

| **Section B: Services, practices and interorganisational collaborations** |
| --- |

- 1. **At your clinic, …**

| 1. is there staff mainly assigned to reception of patients? | 1 Yes | 2  No |
| --- | --- | --- |
| 1. is there staff mainly assigned to manage medical records (opening new files, managing archives)? | 1 Yes | 2  No |
| 1. can a patient leave a message on an answering machine and get a return call from a physician or nurse? | 1 Yes | 2  No |
| 1. at least one doctor make home visits? | 1 Yes | 2  No |
| 1. do you offer services by appointment during weekends (Saturday or Sunday)? | 1 Yes | 2  No |
| 1. do you offer services by appointment during weekday evenings (after 6:00 p.m.)? | 1 Yes | 2  No |
| 1. do you offer walk-in services during weekends (Saturday or Sunday)? | 1 Yes | 2  No |
| 1. do you offer walk-in services during weekday evenings (after 6:00 p.m.)? | 1 Yes | 2  No |
| 1. do you offer services at night (between midnight and 8:00 a.m.)? | 1 Yes | 2  No |
| 1. outside the clinic’s opening hours, do you direct patients to another available clinic? | 1 Yes | 2  No |
| 1. outside the clinic’s opening hours, do you direct patients to the Info-Santé help line? | 1 Yes | 2  No |
| 1. outside the clinic’s opening hours, do you direct patients to hospital emergency departments? | 1 Yes | 2  No |

- 1. **Is your clinic currently accepting new patients for management and follow-up?** Check a single answer only.

| 1 Our clinic accepts all new patients who ask | |
| --- | --- |
| 2 Our clinic accepts new patients BASED ON CERTAIN CONDITIONS only **🢂**Please answer 2.1 | **2.1 What are these conditions?** Check all that apply.  1 Must be a family member of a followed patient at the clinic  2 Must be referred by another doctor  3 Must be a vulnerable patient (as defined by the RAMQ)  4 Must be an orphan patient / registered on an access list  (e.g. guichet d’accès)  5 Other **🢂 Specify**: _______________________________________ |
| 3  Our clinic doesn't accept any new patients | |

- 1. **What percentage of walk-in visits to all visits do you provide at your clinic?**

1 0%  2 1 à 25%  3 26 à 50%  4 51 à 75%  5 76 à 100%

- 1. **To what patients do you offer walk-in services?**

1 All the patients that present

2 Only patients that have a medical record at the clinic (under a doctor’s name)

3 Doesn't apply because we don't offer walk-in services

- 1. **At your clinic, when a patient has an urgent problem, can he or she be seen…**

|  | **Always** | **Often** | **Sometimes** | **Never** |
| --- | --- | --- | --- | --- |
| 1. Between scheduled appointments on the same day? | 1 | 2 | 3 | 4 |
| 1. On the same day during a time slot reserved for emergency cases (e.g. before or after seeing your patients who have appointments)? | 1 | 2 | 3 | 4 |

- 1. **In general, when a patient contacts your clinic, how long does the patient have to wait** (in days) **before seeing a doctor …**

1. in an emergency situation? **_____ days**
2. in a non-emergency situation? **_____ days**
   1. **Does your clinic confirm appointments with patients a few days before scheduled visits?**

1 Yes  2 No

- 1. **When your clinic is closed, is there an on-call system for…**

| 1. vulnerable patients (as defined by the RAMQ)? | 1 Yes | 2  No |
| --- | --- | --- |
| 1. regular patients who have a family doctor at your clinic? | 1 Yes | 2  No |
| 1. people who have a medical record but don’t have a family doctor at your clinic? | 1 Yes | 2  No |
| 1. people who don’t have a medical record at the clinic? | 1 Yes | 2  No |

- 1. **For each client group specified below, indicate if it is possible for patients to contact a physician or nurse by telephone during the clinic’s opening hours?**

| 1. vulnerable patients (as defined by the RAMQ)? | 1 Yes | 2  No |
| --- | --- | --- |
| 1. regular patients who have a family doctor at your clinic? | 1 Yes | 2  No |
| 1. people who have a medical record but don’t have a family doctor at your clinic? | 1 Yes | 2  No |
| 1. people who don’t have a medical record at the clinic? | 1 Yes | 2  No |

- 1. **At your clinic, how much time is scheduled for visits for evaluation of a new patient?** *Check one only.*

1 Less than 10 minutes  4 20 minutes

2 10 minutes_5_ 30 minutes

3 15 minutes  6 Over 30 minutes

1. **At your clinic, how much time is scheduled for follow-up visits?** *Check one only.*

1 Less than 10 minutes  4 20 minutes

2 10 minutes_5_ 30 minutes

3 15 minutes  6 Over 30 minutes

1. **At your clinic, how much time is scheduled for emergency consultations (other than mental disorders)?** *Check one only.*

1 Less than 10 minutes  4 20 minutes

2 10 minutes_5_ 30 minutes

3 15 minutes  6 Over 30 minutes

1. **At your clinic, do you offer systematic patient management and follow-up services for patients who have the following chronic diseases:**
2. diabetes?  1 Yes  2  No
3. chronic obstructive pulmonary disease (COPD)? _1_ Yes  2  No
4. heart failure?  1 Yes  2  No
5. asthma? _1_ Yes  2  No
6. arthritis?  1 Yes  2  No
7. mental disorders?  1 Yes  2  No
8. **At your clinic, do you have …**

|  | **No** | **Yes** | |
| --- | --- | --- | --- |
|  |  | **Computerised** | **Paper** |
| 1. a reminder system to invite patients to have the recommended screening tests (e.g. Pap test)? | 3 | 1 | 2 |
| 1. a checklist in the file concerning the preventive clinical practices (counselling, screening, immunization) to carry out with patients, according to the guidelines that are in effect? | 3 | 1 | 2 |
| 1. a tool to assist lifestyle habit counselling (e.g. for smoking cessation interventions)? | 3 | 1 | 2 |
| 1. a reference tool for services offering support for lifestyle changes (e.g. smoking cessation centre, health education centre)? | 3 | 1 | 2 |
| 1. a chart, in the files of patients with chronic diseases, that includes all the important follow-up components listed in patient management guidelines (e.g. glycated HB in diabetic patients)? | 3 | 1 | 2 |

1. **At your clinic, for follow-up of people with chronic illnesses** (e.g. COPD, diabetes, heart failure, etc.)**, general practitioner(s)…**

|  | **Always** | **Usually** | **Occasionally** | **Rarely** | **Never** |
| --- | --- | --- | --- | --- | --- |
| 1. use a registry to identify and/or track care of patients | 1 | 2 | 3 | 4 | 5 |
| 1. use a tracking system to remind patients about needed visits or services | 1 | 2 | 3 | 4 | 5 |
| 1. follow-up patients between visits by telephone (by the doctor or clinic’s staff) | 1 | 2 | 3 | 4 | 5 |
| 1. use published practice guidelines as the basis for their treatment plans | 1 | 2 | 3 | 4 | 5 |
| 1. involve office staff (administrative or clerical) in identifying and reminding patients in need of follow-up care or other service | 1 | 2 | 3 | 4 | 5 |
| 1. assist patients in setting and attaining self-management goals (e.g. participation of patient in management of their care) | 1 | 2 | 3 | 4 | 5 |
| 1. refer patients to someone **within your practice** for education about their chronic illness | 1 | 2 | 3 | 4 | 5 |
| 1. refer patients to someone **outside your practice** for education about their chronic illness | 1 | 2 | 3 | 4 | 5 |
| 1. use flow sheets in medical records to track critical elements of care | 1 | 2 | 3 | 4 | 5 |

1. **At your clinic, are the following services available?**

| 1. Strep-test | 1 Yes | 2  No |
| --- | --- | --- |
| 1. Skin biopsy | 1 Yes | 2  No |
| 1. IUD insertion | 1 Yes | 2  No |
| 1. Musculo-skeletal injection/aspiration | 1 Yes | 2  No |
| 1. Suture/minor surgery | 1 Yes | 2  No |
| 1. Cervical smear (Pap test) | 1 Yes | 2  No |

1. **Are the following vaccination services offered at your clinic?**

| 1. Childhood vaccination? | 1 Yes | 2  No |
| --- | --- | --- |
| 1. Influenza (seasonal flu) vaccination? | 1 Yes | 2  No |

1. **In your clinic, do any general practitioners provide follow-up for …**

| 1. pregnant women? | 1 Yes | 2 No |  |
| --- | --- | --- | --- |
|  | **🡻**  **18.1 If yes, do they attend delivery?**  1 Yes  2 No | | |
| 1. children aged 5 years or less? | 1 Yes | 2 No |  |
|  | **🡻**  **18.2 If yes, approximately what percentage of  your clinic's clientele does this group represent? __________**% | | |

1. **At your clinic, do any general practitioners focus MOST of their clinical activities or specialize in the following practice field:**

| 1. Delivery attendance and follow-up? | 1 Yes | 2  No |
| --- | --- | --- |
| 1. Women’s health (excluding obstetrical care)? | 1 Yes | 2  No |
| 1. Mental health? | 1 Yes | 2  No |
| 1. Geriatrics? | 1 Yes | 2  No |
| 1. Child and adolescent care? | 1 Yes | 2  No |
| 1. Plastic surgery/treatment of varicose veins? | 1 Yes | 2  No |
| 1. Obesity? | 1 Yes | 2  No |
| 1. One or more chronic diseases in particular (diabetes, COPD, heart failure, etc.)? | 1 Yes | 2  No |
| 1. Industrial medicine/occupational health? | 1 Yes | 2  No |
| 1. Sports medicine? | 1 Yes | 2  No |
| 1. Traveller’s health? | 1 Yes | 2  No |
| 1. Alternative medicine (acupuncture, osteopathy, etc.)? | 1 Yes | 2  No |
| 1. Other? **🢂 Specify**: ____________________________________________________________ | 1 Yes | 2  No |

1. **In addition to the care offered at your clinic, do any of the general practitioners in your clinic also provide care in the following settings or programs:**

| 1. another medical clinic/private office? | 1 Yes | 2  No |
| --- | --- | --- |
| 1. a CLSC (other than yours, if you’re already in a CLSC) for home care services? | 1 Yes | 2  No |
| 1. a CLSC (other than yours, if you’re already in a CLSC) for services other than home care? | 1 Yes | 2  No |
| 1. the emergency room of a general and/or specialized care hospital? | 1 Yes | 2  No |
| 1. a short-term care unit of a general and specialized care hospital? | 1 Yes | 2  No |
| 1. a long-term care facility (CHSLD)*?* | 1 Yes | 2  No |
| 1. medical services provided as part of a palliative care program? | 1 Yes | 2  No |
| 1. obstetrical services in a hospital? | 1 Yes | 2  No |
| 1. other activities identified by the Département régional de médecine générale (DRMG)? | 1 Yes | 2  No |

1. **What are the roles and functions of the nurses on your medical team?** Check all that apply.

| 1 There’s no nurse on our team **🢂**Go to question 22 | 6 Sexually transmitted and blood borne infections (STBI) counselling |
| --- | --- |
| 2 Triage of walk-in patients | 7 Liaison and coordination with CLSC, LTCF, hospitals and other clinics |
| 3 Counselling on tobacco use, diet and physical activity | 8 Support for medical activities (blood pressure, weight, injections, etc.) |
| 4 Health education (e.g. blood glucose testing, blood pressure measurement) | 9 Participation in clinical decisions |
| 5 Follow-up of specific clienteles | 10 Conducting clinical activities as part of a collective prescription |

1. **How is care coordinated among clinic professionals?**

|  | **Always** | **Often** | **Sometimes** | **Never** | **Doesn’t apply because only 1 doctor in the clinic** |
| --- | --- | --- | --- | --- | --- |
| 1. Informal or ad hoc exchanges | 1 | 2 | 3 | 4 | 99 |
| 1. Pre-established care protocols for specific client groups or problems | 1 | 2 | 3 | 4 | 99 |
| 1. Case discussion meetings (statutory meetings) | 1 | 2 | 3 | 4 | 99 |
| 1. Continuing medical education sessions | 1 | 2 | 3 | 4 | 99 |

1. **In the building in which your clinic is located, …**
2. are services offered by medical specialists?

1 Yes **🢂** **If yes, how many different specialities are present?**_________

2 No **🢂** Go to question 24

1. To what degree do the general practitioners in your clinic collaborate (exchange, referrals) with the medical specialists located IN THE SAME BUILDING as your clinic?

1 Quite a bit  2 Somewhat  3 A bit  4 Not at all

1. **To what degree do the general practitioners in your clinic collaborate (exchange, referrals) with medical specialists located ELSEWHERE THAN IN THE BUILDING where your clinic is?**

1 Quite a bit  2 Somewhat  3 A bit  4 Not at all

1. **Where are your clinic’s patients sent when they need to see specialists?**

|  | **Always** | **Often** | **Sometimes** | **Never** |
| --- | --- | --- | --- | --- |
| 1. To a private specialists office | 1 | 2 | 3 | 4 |
| 1. To a hospital or hospital out-patient clinic | 1 | 2 | 3 | 4 |
| 1. To a hospital emergency room | 1 | 2 | 3 | 4 |

1. **In general, appointments with specialists are made by…**

1 the patient  2 clerical staff  3 the clinic nurse  4 the physician

1. **In the building in which your clinic is located, …**
2. are there any services offered by other health professionals (other than physicians)?

1 Yes **🢂 if yes**, **how many different types of other**

**health professionals are present? ___**_________

2  No **🢂** Go to question 28

1. To what degree do the doctors in your clinic collaborate (exchange, referrals) with other health professionals located IN THE SAME BUILDING as your clinic?

1 Quite a bit  2 Somewhat  3 A bit  4 Not at all

1. **To what degree do the doctors in your clinic collaborate (exchange, referrals) with other health professionals (other than physicians) located ELSEWHERE THAN IN THE BUILDING where your clinic is?**

1 Quite a bit  2 Somewhat  3 A bit  4 Not at all

1. **Does your clinic have formal or informal arrangements with other primary healthcare clinics, CLSCs, hospitals and/or medical specialist clinics for any of the following …** *Check all that apply.*

|  | **No** | **Yes** | | | |
| --- | --- | --- | --- | --- | --- |
|  |  | with one or several primary healthcare clinics | with one or several CLSC(s) | with one or several hospital(s) | with one or several specialized clinics |
| 1. planning services offered (on-call activities, clinic office hours, etc.)? | 5 | 1 | 2 | 3 | 4 |
| 1. access to technical services (e.g. radiology, laboratory)? | 5 | 1 | 2 | 3 | 4 |
| 1. exchange of resources (e.g. loan of professionals)? | 5 | 1 | 2 | 3 | 4 |
| 1. follow-up for hospitalised patients or patients seen at the clinic? | 5 | 1 | 2 | 3 | 4 |
| 1. others? **🢂 Specify**: _____________________   _________________________________________ | 5 | 1 | 2 | 3 | 4 |

1. **If you answered “yes” to any of the choices in the preceding question, identify…**
2. **the main primary healthcare clinic or clinics with which you have arrangements:**

_________________________________________________________________________________________________

_________________________________________________________________________________________________

1. **the main CLSC or CLSCs with which you have arrangements:**

_________________________________________________________________________________________________

_________________________________________________________________________________________________

1. **the main hospital or hospitals with which you have arrangements:**

_________________________________________________________________________________________________

_________________________________________________________________________________________________

1. **the main specialized medical clinic or clinics with which you have arrangements:**

_________________________________________________________________________________________________

_________________________________________________________________________________________________

1. **Does your clinic participate in a healthcare access network to ensure that your clinic’s office hours are coordinated with those of other clinics (evenings, weekends, etc.)?**

1 Yes  2 No

1. **Do the general practitioners at your clinic participate in a regional on-call system for vulnerable patients**(as defined by the RAMQ)**?**

1 Yes  2 No

1. **In your clinic, does a general practitioner (or practitioners) participate in …**

| 1. local committees of the *Département régional de médecine générale* *(DRMG)*? | 1 Yes | 2 No |
| --- | --- | --- |
| 1. committees for the implementation of Family Medicine Groups (FMG) and/or Network- Clinics? | 1 Yes | 2 No |
| 1. committees to alleviate congestion in emergency departments? | 1 Yes | 2 No |
| 1. committees on the Health and social service centre’s (CSSS) clinical project? | 1 Yes | 2 No |
| 1. coordination of the «guichet d’accès» for the orphan patients? | 1 Yes | 2 No |
| 1. others **🢂 Specify**: _____________________________________________________ _____________________________________________________________________ | 1 Yes | 2 No |

| **Section C: Vision, mission and value system** |
| --- |

1. **Which statement BEST represents the population that your clinic tries to serve?** Check one only.

1 Anyone who needs services and shows up at the clinic

2 Regular clinic patients or patients registered at the clinic

3 The population in the neighbourhood, village or territory served by the clinic

1. **Using the scale below, indicate how important the following goals are for your clinic:** *Circle your answer choice.*

|  | **More important** | | | | | **Less important** | | | | |
| --- | --- | --- | --- | --- | --- | --- | --- | --- | --- | --- |
| 1. Accessibility of services offered by the clinic | 1 | 2 | 3 | 4 | 5 | 6 | 7 | 8 | 9 | 10 |
| 1. Continuous relationship with patients | 1 | 2 | 3 | 4 | 5 | 6 | 7 | 8 | 9 | 10 |
| 1. Services that meet patients’ physical, psychological and social needs | 1 | 2 | 3 | 4 | 5 | 6 | 7 | 8 | 9 | 10 |
| 1. Delivery of preventive and health promotion services | 1 | 2 | 3 | 4 | 5 | 6 | 7 | 8 | 9 | 10 |
| 1. Services that conform to established guidelines | 1 | 2 | 3 | 4 | 5 | 6 | 7 | 8 | 9 | 10 |
| 1. Respect, courtesy and confidentiality | 1 | 2 | 3 | 4 | 5 | 6 | 7 | 8 | 9 | 10 |
| 1. Equity in health care service delivery and absence of discrimination towards individuals | 1 | 2 | 3 | 4 | 5 | 6 | 7 | 8 | 9 | 10 |
| 1. Improvement of population health | 1 | 2 | 3 | 4 | 5 | 6 | 7 | 8 | 9 | 10 |

1. **Do you *totally agree, partly agree, partly disagree, or totally disagree* with the following statements:**

|  | **Totally agree** | **Partly agree** | **Partly disagree** | **Totally disagree** |
| --- | --- | --- | --- | --- |
| 1. Physicians see the clinic as a business for which financial return is important. | 1 | 2 | 3 | 4 |
| 1. Clinic physicians should consider environmental or occupational causes when assessing patients’ health problems. | 1 | 2 | 3 | 4 |
| 1. Clinic physicians should consider social problems in their clinical interventions for patients (e.g. poverty, violence, substance dependence). | 1 | 2 | 3 | 4 |

1. **Do you *totally agree, partly agree, partly disagree, or totally disagree* with the following statements:**

|  | **Totally agree** | **Partly agree** | **Partly disagree** | **Totally disagree** | **Doesn’t apply because only 1 doctor in the clinic** |
| --- | --- | --- | --- | --- | --- |
| - 1. Clinic professionals share the clinic’s mission, values and objectives. | 1 | 2 | 3 | 4 | 99 |
| - 1. It is important for the clinic’s general practitioners to work as a team. | 1 | 2 | 3 | 4 | 99 |

1. **Choose the statement that corresponds best to your clinic’s vision relating to:**
   - 1. **“responsibility for health”**Check one only.

1 Health is an individual responsibility (it is up to each individual to maintain his or her health or do what it takes to improve his or her health).

2 Health is a collective responsibility (it is up to society to create conditions that help maintain or improve health).

- - 1. **“right to services”** Check one only.

1 Access to care is an absolute right (everyone should have the same access to health care, based on need, regardless of financial ability to pay).

2 Access to care is a relative right (everyone should have access to health care but people who can afford it could pay for better access to health care).

- - 1. **“responsibility / role of physician”** Check one only.

1 The physician is principally a health expert who makes an accurate diagnosis and identifies the most effective treatment for the patient’s disease.

2 The physician is principally a health enabler who uses his/her competence to partner with the patient to take more control over health within the context of the patient’s needs and personal life circumstances.

1. **Choose the one statement that corresponds best to your clinic’s priorities regarding service organization.** Check one only

1 Service accessibility is a higher priority

2 Continuity of care for patients is a higher priority

1. **Choose the one statement that corresponds best to your clinic’s preferred approach for obtaining desired health outcomes for your patients.** Check one only

1 Rather the application of evidence-based medicine and clinical guidelines

2 Rather the participation and empowerment of the patient and his family

| **Section D: Clinic location** |
| --- |

1. **How long has your clinic been in operation?**

1 Less than 1 year _2_ 1 to 4 years _3_ 5 to 9 years _4_ Over 10 years

1. **How long has your clinic been at its current location?**

1 Less than 1 year _2_ 1 to 4 years _3_ 5 to 9 years _4_ Over 10 years

1. **Where is your clinic located?**

1 In a building owned by the physicians or of which they are shareholders

2 In rented offices in a commercial building for health professionals

3 In rented offices in a commercial building for any type of business

4 In an establishment that is part of the publicly-funded health network (hospital, CLSC, etc.)

5 Others 🢂 **Specify:** _______________________________________________________________________________

1. **In the building where your clinic is located, are there other primary healthcare medical teams or other general practitioners who are not part of your clinic?**

1 Yes  2 No

| **Section E: Reorganisation of primary healthcare services** |
| --- |

*Between 2002 and 2005, the ministère de la Santé et des Services sociaux introduced two new health reforms: the creation of Family Medicine Groups (FMG) and the implementation of local services network, under the governance of Health and Social Services Centres (CSSS). We would like to know what you think about these changes. The following questions aim at assessing the changes that have occurred at your clinic in the last five years.*

1. **Since 2005, has there been any change in the medical staff of your clinic?**

1 Yes **🡺** **1.1 If yes how many doctors joined in? ________**

**How many doctors left? ________**

2 No

1. **In your clinic, how have the following activities evolved since 2005?**

|  | **Have**  **INCREASED** | **NO CHANGE** | **Have**  **DECREASED** |
| --- | --- | --- | --- |
| 1. Scope of clinical activities | 1 | 2 | 3 |
| 1. Number of worked hours by doctors | 1 | 2 | 3 |
| 1. Number of walk-in patients seen | 1 | 2 | 3 |
| 1. Medical services offered on weekends | 1 | 2 | 3 |

1. **In your clinic, to what extent have the following elements changed SINCE 2005?**

|  | **IMPROVED** | **NO CHANGE** | **DETERIORATED** |
| --- | --- | --- | --- |
| 1. Working conditions for staff in your clinic | 1 | 2 | 3 |
| 1. Administrative support in your clinic | 1 | 2 | 3 |
| 1. Clinical practice support for general practitioners in your clinic | 1 | 2 | 3 |
| 1. Quality of care delivered to patients in your clinic | 1 | 2 | 3 |
| 1. Your clinic’s access to lab/imaging facility («plateaux techniques») | 1 | 2 | 3 |
| 1. The possibility of having one or several nurses in your clinic | 1 | 2 | 3 |
| 1. Collaboration between your clinic and other primary care clinics in your territory | 1 | 2 | 3 |
| 1. The ease by which your patients can be seen by specialists | 1 | 2 | 3 |
| 1. Collaboration between your clinic and the CSSS | 1 | 2 | 3 |
| 1. Collaboration between your clinic and hospitals outside the CSSS | 1 | 2 | 3 |
| 1. The possibility of recruiting new physicians to your clinic | 1 | 2 | 3 |
| 1. Teamwork among professionals from your clinic | 1 | 2 | 3 |
| 1. Access to information technologies | 1 | 2 | 3 |
| 1. Level of financial resources available for your clinic | 1 | 2 | 3 |

1. **How would you assess the effect of the following on your clinic since 2005?**

|  | **Very positive** | **Positive** | **No effect** | **Negative** | **Very negative** |
| --- | --- | --- | --- | --- | --- |
| 1. Role played by the local DRMG | 1 | 2 | 3 | 4 | 5 |
| 1. Actions taken by the CSSS | 1 | 2 | 3 | 4 | 5 |
| 1. Involvement of the Ordre des infirmières et des infirmiers du Québec | 1 | 2 | 3 | 4 | 5 |
| 1. Involvement of the Collège des médecins | 1 | 2 | 3 | 4 | 5 |
| 1. Involvement of regional FMOQ representatives and affiliated local associations | 1 | 2 | 3 | 4 | 5 |
| 1. Introduction of FMG in your region | 1 | 2 | 3 | 4 | 5 |
| 1. Introduction of Network-Clinics in your region | 1 | 2 | 3 | 4 | 5 |
| 1. Measures associated with AMP (activités médicales particulières) | 1 | 2 | 3 | 4 | 5 |
| 1. Measures associated with PREM (plan régional d’effectifs médicaux) | 1 | 2 | 3 | 4 | 5 |
| 1. Exemplary practices in other primary care clinics | 1 | 2 | 3 | 4 | 5 |

1. **In the territory where your clinic is located, is there a medical clinic** (including your own) **that you think of as a model to emulate?**

1 Yes **🢂** **5.1 What is the name of this medical clinic?** _________________________________________________

2 No

1. **The current status of your clinic is…**

| 1. FMG-main site | 1 Yes **🢂** | **6.1 If yes, are all physicians in your clinic part of if?**  1 Yes  2 No | |
| --- | --- | --- | --- |
|  | 2 No |  |  |
| 1. FMG-affiliated site | 1 Yes **🢂** | **6.2 If yes, are all physicians in your clinic part of if?**  1 Yes  2 No | |
|  | 2 No |  |  |
| 1. Network-Clinic | 1 Yes |  |  |
|  | 2 No |  |  |

1. **If your clinic has become a FMG and/or Network-Clinic, to what extent did the following reasons encouraged you to do so?**

|  | **Highly** | **Fairly** | **Slightly** | **Not at all** |
| --- | --- | --- | --- | --- |
| - - 1. To increase human resources at the clinic | 1 | 2 | 3 | 4 |
| - - 1. To increase technological resources at the clinic | 1 | 2 | 3 | 4 |
| - - 1. To improve the clinic’s image | 1 | 2 | 3 | 4 |
| - - 1. To increase the clinic’s clientele | 1 | 2 | 3 | 4 |
| - - 1. To optimise the clinic’s profitability | 1 | 2 | 3 | 4 |
| - - 1. To recruit new physicians | 1 | 2 | 3 | 4 |
| - - 1. To improve the quality of care and services for clinic patients | 1 | 2 | 3 | 4 |
| - - 1. To improve access to care for people in your territory | 1 | 2 | 3 | 4 |
| - - 1. To enhance physicians’ access to lab/imaging facility («plateaux techniques») | 1 | 2 | 3 | 4 |
| - - 1. To develop multidisciplinarity | 1 | 2 | 3 | 4 |
| - - 1. To release physicians from certain tasks | 1 | 2 | 3 | 4 |

1. **If your clinic is neither an FMG nor a Network-Clinic, do you intend on becoming...**

| - - 1. an FMG-main site? | 1 Yes | 2  No |
| --- | --- | --- |
| - - 1. an FMG-affiliated site? | 1 Yes | 2  No |
| - - 1. a Network-Clinic? | 1 Yes | 2  No |
| - - 1. another type of organisation **🢂 Specify:** ________________________________________________   ________________________________________________ | 1 Yes | 2  No |

1. **If applicable, to what extent do the following reasons encourage your clinic to become an FMG, Network-Clinic or both?**

|  | **Highly** | **Fairly** | **Slightly** | **Not at all** |
| --- | --- | --- | --- | --- |
| 1. To increase human resources at the clinic | 1 | 2 | 3 | 4 |
| 1. To increase technological resources at the clinic | 1 | 2 | 3 | 4 |
| 1. To improve the clinic’s image | 1 | 2 | 3 | 4 |
| 1. To increase the clinic’s clientele | 1 | 2 | 3 | 4 |
| 1. To optimise the clinic’s profitability | 1 | 2 | 3 | 4 |
| 1. To recruit new physicians | 1 | 2 | 3 | 4 |
| 1. To improve the quality of care and services for clinic patients | 1 | 2 | 3 | 4 |
| 1. To improve access to care for people in your territory | 1 | 2 | 3 | 4 |
| 1. To facilitate physicians’ access to technical support centres | 1 | 2 | 3 | 4 |
| 1. To develop multidisciplinarity | 1 | 2 | 3 | 4 |
| 1. To release physicians from certain tasks | 1 | 2 | 3 | 4 |

1. **If applicable, why doesn’t your clinic intend to become a FMG and/or Network-Clinic?** Check all that apply.

1 Clinic’s current situation not favourable to such changes

2 Procedure cumbersome and complex

3 Lack of support, information and/or guidance

4 Fear of a loss of autonomy

5 Doesn’t meet the criteria set out by the MSSS and/or Agence de santé et services sociaux

6 Others **🢂Specify*:* ______________________________________________________________________________**

**END OF THE QUESTIONNAIRE**

THANK YOU FOR YOUR COLLABORATION!

If you have any additional comments, please write them down in the space provided below.

They will be examined with great attention.

_______________________________________________________________________________________________________

_______________________________________________________________________________________________________

_______________________________________________________________________________________________________

_______________________________________________________________________________________________________

_______________________________________________________________________________________________________

_______________________________________________________________________________________________________

_______________________________________________________________________________________________________

_______________________________________________________________________________________________________

_______________________________________________________________________________________________________

_______________________________________________________________________________________________________

_______________________________________________________________________________________________________

_______________________________________________________________________________________________________

_______________________________________________________________________________________________________

_______________________________________________________________________________________________________

_______________________________________________________________________________________________________

_______________________________________________________________________________________________________

_______________________________________________________________________________________________________

_______________________________________________________________________________________________________

**Date: ______/_____/______**

**(Day /Month/Year)**

March 2010

**PRINCIPAL INVESTIGATORS**

Jean-Frédéric Levesque

Raynald Pineault

Pierre Tousignant

**COLLABORATION**

The research project's team of researchers and associated collaborators

**INSTITUTIONAL SUPPORT**

(1) Canadian Institutes of Health Research (CIHR); (2) Fonds de recherche en santé du Québec (FRSQ); (3) Ministère de la Santé et des Services sociaux du Québec; (4) Agence de la santé et des services sociaux de Montréal – Direction de santé publique; (5) Agence de la santé et des services sociaux de la Montérégie; (6) Institut national de santé publique du Québec (INSPQ).

This project has been approved by the Comité d'éthique de la recherche de l'Agence de la santé et des services sociaux de Montréal.

* Use of this questionnaire, in whole or in part, is not permitted without the authorization of the lead researchers.
