# Supplementary material for: Assessing the evolution of primary healthcare organizations and their performance (2005-2010) in two regions of Québec province: Montréal and Montérégie
Source: BMC Fam Pract. 2010 Dec 1;11:95. doi: 10.1186/1471-2296-11-95 (PMC3014883; doi:10.1186/1471-2296-11-95)
Supplement: Additional file 5 — Population Questionnaire. This file contains the questionnaire used in the population survey. Questions relate mainly to experience of care, utilization and unmet needs. [file 1471-2296-11-95-S5.DOC]

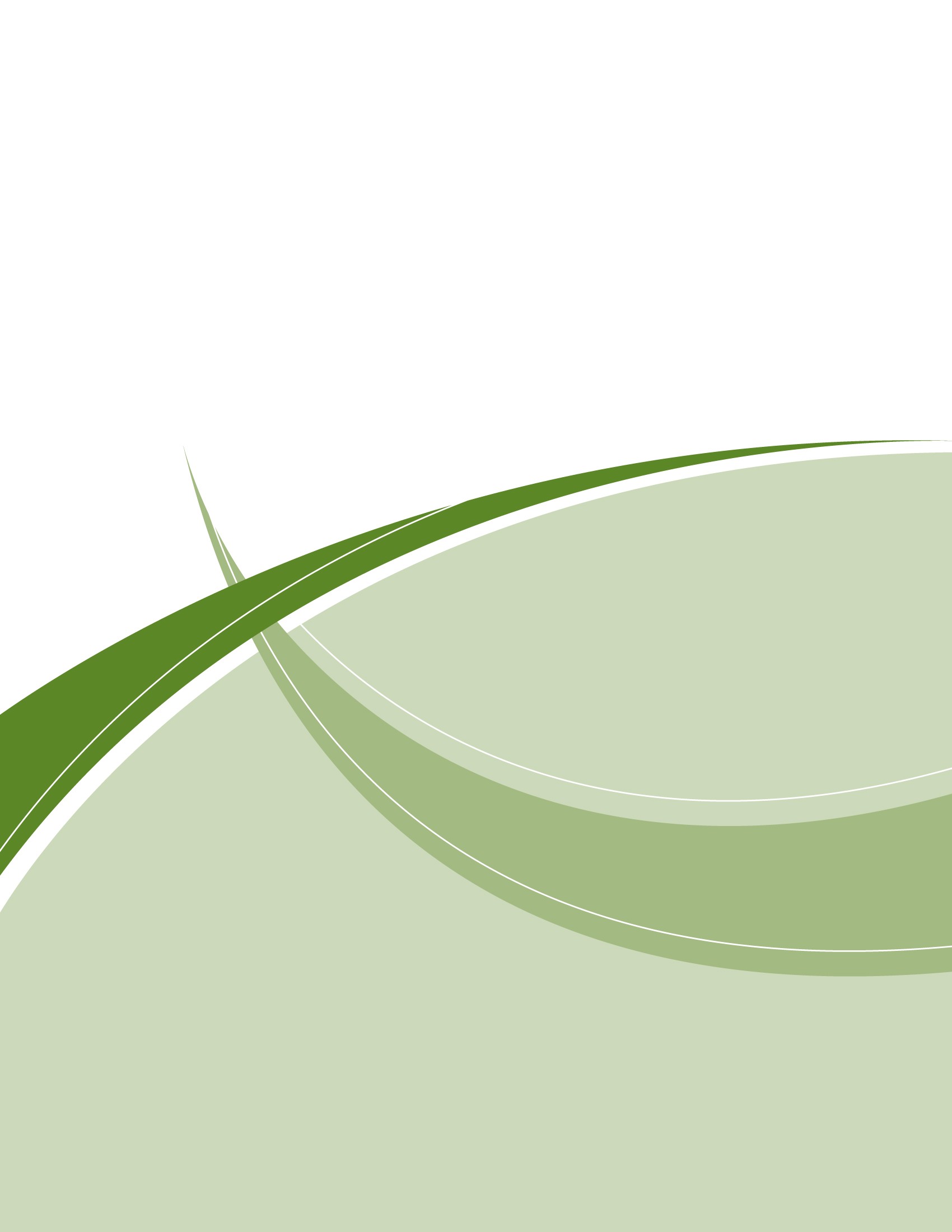

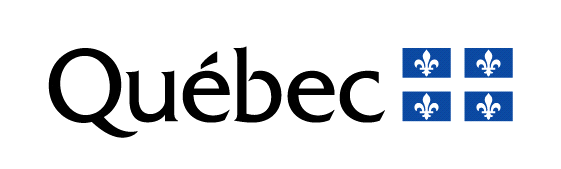
**Assessing the evolution of primary healthcare organizations**

**and their performance (2005-2010) in two regions of**

**Québec province: Montréal and Montérégie**

**Additional file 5 :**

**Population Questionnaire**

July 21, 2010

Institut national de santé publique du Québec

Direction de santé publique, Agence de la santé et des services sociaux de Montréal

**Components of the questionnaire**

**Section A Health services utilization**………………………………………………………………………p. 3

Utilization profile over the last two years

Identification of usual source of care

Family doctor

**Section B Primary care experience with the usual source of care**……………………………………….p. 6

Accessibility

Organizational accessibility

Comprehensiveness

Continuity of affiliation and follow-up

Responsiveness

Outcomes of services

Informational continuity

**Section C Unmet needs for care**……………………………………………………………………………p. 12

**Section D Health status**......................………………………………………………………..…………..p. 14

**Section E Clinical preventive services** .……………………………………………………..………..…...p. 16

**Section F Sociodemographic characteristics**………………………………………………………………p. 20

**Section A – Health services utilization**

- **Utilization profile over the last two years**

A1 In the last two years, since 2008, how many times were you hospitalised (you spent at least one night in the hospital)?

❑ 0 never

❑ 1 once

❑ 2 twice

❑ 3 3 times or more

❑ 8 doesn’t know/doesn’t remember

A2 In the last two years, how many times did you go to a hospital emergency room to get medical care?

❑ 0 never

❑ 1 once

❑ 2 twice

❑ 3 3 times

❑ 4 4 times

❑ 5 5 times or more

❑ 8 doesn’t know/doesn’t remember

A3 In the last two years, did you go to a CLSC to see a doctor?

❑ 1 yes

❑ 2 no

❑ 8 doesn’t know/doesn’t remember

A4 In the last two years, did you go to a doctor’s office or medical clinic to see a general practitioner or a family physician (other than the CLSC)? (including regular check-ups and follow-ups or for a form to be filled)

*(Note : including UMF (Family Medicine Unit))*

❑ 1 yes

❑ 2 no

❑ 8 doesn’t know/doesn’t remember

- If the respondent did not use medical services in a CLSC or a medical clinic (A3 = 2 or 8 and A4 = 2 or 8), *ask A4B then go to A11*

A4B Are you followed by a medical specialist?

❑ 1 yes

❑ 2 no

❑ 8 doesn’t know/doesn’t remember

**Identification of usual source of care**

The next questions refer to the place you usually go to or the doctor you usually see when you need general medical care. All information is anonymous and confidential, and no personal information will be given to a clinic or doctor.

A5 In the last two years, where did you usually go to see a doctor for your general medical care, excluding specialised care? Was it…

❑ 1 at a clinic or doctor’s office (including Family Medicine Groups or FMG) *Go to Q_S1*

❑ 2 at a CLSC *Go to Q_S1*

❑ 3 at an FMU clinic (Family Medicine Unit in a hospital) *Go to Q_S1*

❑ 4 at a hospital emergency room *Go to A11*

❑ 5 at a doctor’s office in a seniors residence *Go to A11*

❑ 6 homecare *Go to A11*

❑ 7 no usual place for general medical care services *Go to A5B*

❑ 97 others (Specify) *Go to A11* ________________________________

❑ 98 doesn’t know/doesn’t remember *Go to A11*

A5B In the last two years, where did you go to most often for your general medical care? (At least once) (In case of multiple answers, select the one that is most important for the respondent.) (As a last resort, select the most recent.)

❑ 1 at a clinic or doctor’s office (including Family Medicine Groups or FMG)

❑ 2 at a CLSC

❑ 3 at an FMU clinic (Family Medicine Unit in a hospital)

❑ 4 at a hospital emergency room *Go to A11*

❑ 5 at a doctor’s office in a seniors residence *Go to A11*

❑ 6 homecare *Go to A11*

❑ 97 others (Specify) *Go to A11* ________________________________

❑ 98 doesn’t know/doesn’t remember *Go to A11*

Clinic identification

**Q_S1** Could you please tell me the name of your clinic (your usual source of health care)? ____________________________

We are now going to talk only about the **“*Name of the source of care*”** to get a better idea of the services available.

A6 How long have you been going to this place?

❑ 1 less than 2 years

❑ 2 2 to 5 years

❑ 3 6 to 9 years

❑ 4 10 years or more

❑ 8 doesn’t know/doesn’t remember

A7 In the last two years, how many times did you go to this place to seek care for yourself? (Approximately, about)

❑ 1 once

❑ 2 2 to 5 times

❑ 3 6 to 9 times

❑ 4 more than10 times

❑ 8 doesn’t know/doesn’t remember

A8 At this place, do you have a family doctor?

❑ 1 yes *Go to B1*

❑ 2 no

❑ 8 doesn’t know/doesn’t remember

A9 (At this place), is there a particular doctor who takes principal responsibility for your care (without officially being your family doctor)?

❑ 1 yes *Go to B1*

❑ 2 no

❑ 8 doesn’t know/doesn’t remember

A10 Do you have a family doctor that you see in another clinic or health institution?

❑ 1 yes *Go to B1*

❑ 2 no *Go to A13*

❑ 8 doesn’t know/doesn’t remember *Go to B1*

**For people who did not use services in the past two years and for respondents who don’t have a regular source of care:**

A11 Do you have a family doctor?

❑ 1 yes

❑ 2 no *Go to A13*

❑ 8 doesn’t know/doesn’t remember *Go to C1*

A12 Do you usually see your family doctor…

❑ 1 at a clinic or doctor’s office

❑ 2 at a CLSC

❑ 3 at a FMU clinic (family medicine unit in a hospital)

❑ 4 at a hospital emergency room

❑ 5 at a doctor’s office in a seniors residence

❑ 6 homecare

❑ 97 others

❑ 98 doesn’t know/doesn’t remember

*Go to C1*

❑ 95 does not know

A13 Why don’t you have a family doctor? Is it because... (*main reason*)

❑ 1 you don't want one, or you’ve never tried to get one, or you don’t need one

❑ 2 none are available or are taking new patients in your region

❑ 3 you had one that left or retired

❑ 4 you moved and lost your family doctor

❑ 5 where you go, you can’t have a regular physician

❑ 6 you’re being followed by one or several specialists

❑ 97 others, *Specify* __________________________________________

❑ 98 doesn’t know/doesn’t remember

- If the respondent is a non user of services (A3 = 2 or 9 and A4 = 2 or 9) or doesn’t have a regular source of care in a medical clinic (A5 ≥ 4 and A5B ≥ 4)*, go to C1*
- If not, *go to B1*

**Section B - Primary care experience with the usual source of care**

**Accessibility**

For each of the following statements, keeping in mind your experience over the past two years, tell me if the statements apply always, often, sometimes or never to the («Name of the source of care») where you usually go for health care.

|  |  | Always | Often | Sometimes | Never | Doesn’t know/Doesn’t remember |
| --- | --- | --- | --- | --- | --- | --- |
| B1 | When you go to this place, you see the same doctor | 1 | 2 | 3 | 4 | 8 |
| B2 | At this place, if the doctor who is responsible for your care is not available, you can see another doctor (*Additional response choice: «7 – Not applicable» if only one doctor in the clinic)* | 1 | 2 | 3 | 4 | 8 |
| B3 | If you need to see a doctor for a new health problem, you go to this place first | 1 | 2 | 3 | 4 | 8 |
| B4 | If you need to see a doctor on the same day for a health problem, you go to this place first | 1 | 2 | 3 | 4 | 8 |
| B5 | When you consult a doctor at this place, you go directly there without making an appointment | 1  *Go to B10* | 2 | 3 | 4 | 8 |

B6 When you need to see a doctor at this place, in general, how long does it take to see the doctor by appointment?

❑ 1 less than 2 weeks

❑ 2 2 to 4 weeks

❑ 3 1 to 3 months

❑ 4 4 months or more

❑ 5 pre-scheduled appointments *Go to B8*

❑ 6 never takes an appointment *Go to B10*

❑ 8 doesn’t know/doesn’t remember *Go to B8*

B7 Would you say that this delay before you can see a doctor by appointment is…

❑ 1 very long

❑ 2 long

❑ 3 short

❑ 4 very short

❑ 8 doesn’t know/doesn’t remember

B8 When you have an appointment at this place, in general, how long do you have to wait between the scheduled time of appointment and the time you actually see the doctor?

❑ 1 less than 30 minutes

❑ 2 30 to 59 minutes

❑ 3 1 hour or more

❑ 8 doesn’t know/doesn’t remember *Go to B10*

B9 Would you say that this waiting period is…

❑ 1 very long

❑ 2 long

❑ 3 short

❑ 4 very short

❑ 8 doesn’t know/doesn’t remember

B10 When you need immediate or emergency care, how long does it take to see the doctor at this place?

❑ 1 less than 24 hours

❑ 2 1 to 2 days

❑ 3 3 to 4 days

❑ 4 5 days or more

❑ 8 doesn’t know/doesn’t remember

*Note : Ask B11 only to the respondents who answered Yes at A8 or A9 (A8 = 1 or A9 = 1).*

B11 During the past two years, did you contact the («Name of the source of care») to see your doctor on the same day or the next day and your doctor was not available by appointment?

❑ 1 yes

❑ 2 no *Go to B12*

❑ 8 doesn’t know / doesn’t remember *Go to B12*

| On these situations, are you given the option to… | | Always | Often | Sometimes | Never | Doesn’t know/Doesn’t remember |
| --- | --- | --- | --- | --- | --- | --- |
| B11A | come to the walk-in clinic (*Additional response choice: «7 – Not applicable» if no walk-in in the clinic).* | 1 | 2 | 3 | 4 | 8 |
| B11B | have a doctor or nurse call you back | 1 | 2 | 3 | 4 | 8 |
| B11C | see another doctor by appointment (*Additional response choice: «7 – Not applicable» if only one doctor in the clinic).* | 1 | 2 | 3 | 4 | 8 |
| B11D | see your doctor between two patients | 1 | 2 | 3 | 4 | 8 |
| B11E | go to another walk-in clinic or to the emergency room | 1 | 2 | 3 | 4 | 8 |

B12 Would you say that the geographic location of the («Name of the source of care») in relation to where you live is…

❑ 1 very close

❑ 2 close

❑ 3 far

❑ 4 very far

❑ 8 doesn’t know/doesn’t remember

B13 How long does it usually take you to get there?

❑ 1 less than 15 minutes

❑ 2 15 to 30 minutes

❑ 3 more than 30 minutes

❑ 8 doesn’t know/doesn’t remember

When you go to the («Name of the source of care»), tell me if the following statements apply always, often, sometimes or never.

|  | | Always | Often | Sometimes | Never | Doesn’t know/Doesn’t remember |
| --- | --- | --- | --- | --- | --- | --- |
| B14 | When you go to this place, you lose income or study time, or you miss classes | 1 | 2 | 3 | 4 | 8 |
| B15 | You have to pay a fee for having a file opened or kept up to date, such as administrative fees, annual fees, etc. | 1 | 2 | 3 | 4 | 8 |
| B16 | You have to pay for laboratory tests or radiology exams done on site or somewhere else (blood tests, X-ray, scan or ultrasound, etc.) | 1 | 2 | 3 | 4 | 8 |
| B17 | You have to pay for OTHER doctor’s services that are not covered by the Régie de l’assurance-maladie, for example, to pay for the consultation or to get the doctor to fill out a form for you (even if rembursed by private insurance) | 1 | 2 | 3 | 4 | 8 |
| B18 | You have to pay for drugs given on-site like eye drops, local anesthetics or medical supplies like bandages, syringes, etc. | 1 | 2 | 3 | 4 | 8 |
| B19 | You have to pay other costs, when you go to this place | 1 | 2 | 3 | 4 | 8 |

*If the answer is «4 –Never» at questions B14 to B19, go to B21*

B20 Do you consider that the costs related to the care received at (“Nom de la source de soins”) are …?

❑ 1 very high

❑ 2 high

❑ 3 moderate

❑ 4 low

❑ 5 very low

❑ 8 doesn’t know/doesn’t remember

We would now like to know your opinion about how accessible the («Name of the source of care») is. For each of the following statements tell me if you agree strongly, somewhat, a little or not at all.

|  |  | Strongly agree | Somewhat | A little | Not at all agree | Doesn’t know/Doesn’t remember |
| --- | --- | --- | --- | --- | --- | --- |
| B21 | The office hours are convenient | 1 | 2 | 3 | 4 | 8 |
| B22 | It is easy to reach someone at this place by telephone to make an appointment | 1 | 2 | 3 | 4 | 8 |
| B23 | It is easy to get a appointment at this place | 1 | 2 | 3 | 4 | 8 |
| B24 | It is easy to talk to a doctor or nurse by telephone when this place is open | 1 | 2 | 3 | 4 | 8 |
| B25 | It is easy to reach a doctor or nurse by telephone even when this place is closed | 1 | 2 | 3 | 4 | 8 |

**Comprehensiveness**

Still keeping in mind your experience over the last two years, tell me if you agree strongly, somewhat, a little or not at all with each of the following statements.

| *At this place...* | | Strongly agree | Somewhat | A little | Not at all agree | Doesn’t know/Doesn’t remember |
| --- | --- | --- | --- | --- | --- | --- |
| B27 | all your health problems are taken care of, whether they are physical or psychological | 1 | 2 | 3 | 4 | 8 |
| B28 | during your visits, the doctor takes the time to talk to you about prevention and asks you about your lifestyle habits | 1 | 2 | 3 | 4 | 8 |
| B29 | at this place, they help you get all the health care services you need | 1 | 2 | 3 | 4 | 8 |
| B30 | your opinion and what you want are taken into account in the care that you receive | 1 | 2 | 3 | 4 | 8 |
| B31 | you are given help to weigh the pros and cons when you have to make decisions about your health | 1 | 2 | 3 | 4 | 8 |

**Continuity of affiliation and follow-up**

| *At this place…* | | Strongly agree | Somewhat | A little | Not at all agree | Doesn’t know/Doesn’t remember |
| --- | --- | --- | --- | --- | --- | --- |
| B32 | your medical history is known (your past medical history) | 1 | 2 | 3 | 4 | 8 |
| B33 | they are aware of all the prescribed medications you take *(Additional response choice: «7- doesn’t take prescribed medications»)* | 1 | 2 | 3 | 4 | 8 |
| B34 | you can receive routine ongoing care for a chronic problem, for example, for high blood pressure, diabetes or back pain | 1 | 2 | 3 | 4 | 8 |

**Responsiveness**

| *At this place…* | | Strongly agree | Somewhat | A little | Not at all agree | Doesn’t know/Doesn’t remember |
| --- | --- | --- | --- | --- | --- | --- |
| B35 | clinic staff answer your questions clearly | 1 | 2 | 3 | 4 | 8 |
| B36 | you feel respected | 1 | 2 | 3 | 4 | 8 |
| B37 | you are greeted courteously at the reception | 1 | 2 | 3 | 4 | 8 |
| B38 | your physical privacy is respected | 1 | 2 | 3 | 4 | 8 |
| B39 | the doctors spend enough time with you | 1 | 2 | 3 | 4 | 8 |
| B40 | the premises are pleasant | 1 | 2 | 3 | 4 | 8 |

**Outcomes of care**

Keeping in mind the services and care you have received there, tell me if you agree strongly, somewhat, a little or not at all with each of the following statements.

|  | | Strongly agree | Somewhat | A little | Not at all agree | Doesn’t know/Doesn’t remember |
| --- | --- | --- | --- | --- | --- | --- |
| B41 | The services you get there help you…  …better understand your health problems | 1 | 2 | 3 | 4 | 8 |
| B42 | …prevent certain health problems before they appear | 1 | 2 | 3 | 4 | 8 |
| B43 | …control your health problems | 1 | 2 | 3 | 4 | 8 |
| B44 | The professionals you see there encourage you to follow the treatments prescribed | 1 | 2 | 3 | 4 | 8 |
| B45 | The professionals you see there help motivate you to adopt good lifestyle habits like quitting smoking, eating better, etc. | 1 | 2 | 3 | 4 | 8 |

**Informational continuity**

The following questions are about other services you might have received.

B46 During the past two years, did you go for any lab tests or radiological examinations prescribed by a doctor at («Name of the source of care»)? (Labs tests : blood or urine tests ; Radiological examinations : X-ray or ultrasound. Radiological exams also include: scanner, magnetic resonance, Doppler, scintigraphy, colonoscopy, mammography, etc.)

❑ 1 yes

❑ 2 no *Go to B50*

❑ 8 doesn’t know/doesn’t remember *Go to B50*

Regarding these tests or exams, tell me if the following statements apply always, often, sometimes or never.

|  |  | Always | Often | Sometimes | Never | Doesn’t know/Doesn’t remember |
| --- | --- | --- | --- | --- | --- | --- |
| B47 | Someone at («Name of the source of care») called you or met with you to give you the results of these tests or exams | 1 | 2 | 3 | 4 | 8 |
| B48 | The doctor at the clinic was aware of your tests or exams results when you saw him or her again at the clinic *(Additional response choice: « 7 - hasn’t seen the doctor again yet» Go to B50)* | 1 | 2 | 3 | 4 | 8 |
| B49 | You were given clear explanations about the results of your tests or exams | 1 | 2 | 3 | 4 | 8 |

B50 During the past two years, did you see one or more specialists to whom you were referred by a doctor at ("Name of the source of care”)?

❑ 1 yes

❑ 2 no *Go to B53*

❑ 8 doesn’t know/doesn’t remember *Go to B53*

Regarding your visits to this or these specialists, tell me if…

|  |  | Always | Often | Sometimes | Never | Doesn’t know/Doesn’t remember |
| --- | --- | --- | --- | --- | --- | --- |
| B51 | The doctor at the clinic was aware of the results of your visit to a specialist when you saw him or her again at the clinic *(Additional response choice: «7 - hasn’t seen the doctor again yet» Go to B53)* | 1 | 2 | 3 | 4 | 8 |
| B52 | After you saw the specialist(s), a doctor at the clinic discussed the specialist’s report with you | 1 | 2 | 3 | 4 | 8 |

B53 During the past two years, did you see one or more health professionals to whom you were referred by a doctor at («Name of the source of care») (e.g. nutritionist, physiotherapist)?

❑ 1 yes

❑ 2 no *Go to B56*

❑ 8 doesn’t know/doesn’t remember *Go to B56*

Regarding your visits to this or these health professionals, tell me if…

|  |  | Always | Often | Sometimes | Never | Doesn’t know/Doesn’t remember |
| --- | --- | --- | --- | --- | --- | --- |
| B54 | The doctor at the clinic was aware of the results of these visits when you saw him or her again at the clinic *(Additional response choice: «7 - hasn’t seen the doctor again yet» Go to note before B56 )* | 1 | 2 | 3 | 4 | 8 |
| B55 | After your visits to this or these health professionals, a doctor at the clinic discussed the professional’s report with you | 1 | 2 | 3 | 4 | 8 |

**Note**: *Ask B56 only to respondents who answered Yes to B46 or B50 or B53*

|  |  | Always | Often | Sometimes | Never | Doesn’t know/Doesn’t remember |
| --- | --- | --- | --- | --- | --- | --- |
| B56 | Someone at («Name of the source of care») helped you take appointments for tests, to see a specialist or for assessments with other health professionals | 1 | 2 | 3 | 4 | 8 |

**Section C – Unmet needs for care**

The next questions are about your needs for health services.

C1 During the last 6 months, did you feel you needed to see a doctor for a health problem but didn’t see one?

❑ 1 yes

❑ 2 no *Go to D1*

❑ 8 doesn’t know/doesn’t remember *Go to D1*

C2 For what reason did you want to see a doctor? Was it...

*(Note: If several unmet needs for services, respond according to the most important)*

❑ 1 for an urgent health problem

❑ 2 for a non-urgent health problem

❑ 3 to have routine exams or follow-up (including prescription renewal) *Go to C8*

❑ 4 to receive results of tests, exams, or consultation to a specialist *Go to C8*

❑ 5 to have a form filled out *Go to C8*

❑ 97 other reason *Go to C8*

❑ 98 doesn’t know/doesn’t remember *Go to C8*

C2.1 Was this a known problem or was it a new problem?

*(Note: Known by the respondent himself)*

❑ 1 known problem

❑ 2 new problem

❑ 8 doesn’t know/doesn’t remember

C3 Do you still have this problem?

❑ 1 yes

❑ 2 no

❑ 8 doesn’t know/doesn’t remember

Tell me if the following statements apply a lot, moderately, slightly or not at all to your situation.

|  |  | A lot | Moderately | Slightly | Not at all | Doesn’t know/Doesn’t remember |
| --- | --- | --- | --- | --- | --- | --- |
| C4 | This problem was causing you pain | 1 | 2 | 3 | 4 | 8 |
| C5 | This problem was threatening your health | 1 | 2 | 3 | 4 | 8 |
| C6 | You were afraid of having complications if this problem was not treated quickly | 1 | 2 | 3 | 4 | 8 |
| C7 | The problem for which you wanted to consult was limiting your activities | 1 | 2 | 3 | 4 | 8 |

We would like to know the reasons why you didn’t see a doctor. I will read you a list of reasons. Tell me if they apply to your situation.

|  |  | Yes | No | Doesn’t know/Doesn’t remember |
| --- | --- | --- | --- | --- |
| C8 | You have an appointment but have not seen the doctor yet | 1  *Go to C18* | 2 | 8 |
| C9 | You didn’t know where to go to see a doctor | 1 | 2 | 8 |
| C10 | You couldn’t get an appointment | 1 | 2 | 8 |
| C11 | You couldn’t move around to actually go see a doctor | 1 | 2 | 8 |
| C12 | You couldn’t find a doctor who is taking new patients | 1 | 2 | 8 |
| C13 | Your usual doctor wasn’t available at the time you needed him or her | 1 | 2 | 8 |
| C14 | The waiting time before seeing a doctor was too long | 1 | 2 | 8 |
| C15 | The office hours during which you could see a doctor did not suit you | 1 | 2 | 8 |
| C16 | Your health status deteriorated too much so that you could not go see a doctor | 1 | 2 | 8 |
| C17 | Your problem resolved by itself | 1  *Go to C20* | 2 | 8 |

Still thinking about the problem for which you wanted to see a doctor, tell me if you agree strongly, somewhat, a little or not at all with each of the following statements. Since the problem appeared…

*Note: conditional to C2 = 1 or 2, if not go to section D*

|  |  | Strongly | Somewhat | A little | Not at all | Doesn’t know/Doesn’t remember |
| --- | --- | --- | --- | --- | --- | --- |
| C18 | Your health has worsened | 1 | 2 | 3 | 4 | 8 |
| C19 | You still have not succeeded in managing your health problem | 1 | 2 | 3 | 4 | 8 |
| C20 | This situation caused you to feel worried, stressed or anxious | 1 | 2 | 3 | 4 | 8 |
| C21 | This situation caused some of your family members and friends to feel worried, stressed or anxious | 1 | 2 | 3 | 4 | 8 |
| C22 | This situation had negative consequences on other aspects of your life | 1 | 2 | 3 | 4 | 8 |
| C23 | You lost some income | 1 | 2 | 3 | 4 | 8 |
| C24 | This situation made you dependent on family members or friends | 1 | 2 | 3 | 4 | 8 |

**Section D – Health status**

We still have a few questions about your health status and prevention.

**Health status**

D1 In general, would you say your health is...

❑ 1 excellent

❑ 2 very good

❑ 3 good

❑ 4 average

❑ 5 poor

❑ 8 doesn’t know/doesn’t remember

D2 Can I please confirm that you are a…

❑ 1 male

❑ 2 female

*(Note : this question is asked at the beginning of the interview)*

D3 How old are you?

The following questions are about current restrictions in your daily activities caused by a health problem or condition that has lasted or will last 6 months or more.

| Are you limited in your daily activities due to… | | Always | Often | Sometimes | Never | Doesn’t know/Doesn’t remember |
| --- | --- | --- | --- | --- | --- | --- |
| D4 | … difficulty seeing, hearing or communicating? | 1 | 2 | 3 | 4 | 8 |
| D5 | … difficulty walking, going up stairs or bending over? | 1 | 2 | 3 | 4 | 8 |

The next questions are about various health problems. We would like to remind you that this information is going to be treated anonymously.

D6 Has a doctor ever told you that you have high blood pressure or hypertension?

❑ 1 yes

❑ 2 no *Go to D8*

❑ 8 doesn’t know/doesn’t remember

D7 Do you take prescription drugs for high blood pressure?

❑ 1 yes

❑ 2 no

❑ 8 doesn’t know/doesn’t remember

D8 *(Has a doctor ever told you that you have)* diabetes (sugar in the blood or urine)?

❑ 1 yes

❑ 2 no *Go to D10*

❑ 8 doesn’t know/doesn’t remember

D9 Do you take prescription drugs for diabetes?

❑ 1 yes

❑ 2 no

❑ 8 doesn’t know/doesn’t remember

D10 *(Has a doctor ever told you that you have)* high level of cholesterol?

❑ 1 yes

❑ 2 no *Go to D12*

❑ 8 doesn’t know/doesn’t remember

D11 Do you take prescription drugs to lower your cholesterol?

❑ 1 yes

❑ 2 no

❑ 8 doesn’t know/doesn’t remember

D12 *(Has a doctor ever told you that you have)* a heart disease or heart problem? (Read the following list if needed : angina, infarct, arrhythmia, prior heart operation, heart failure, water on the lungs, …)

❑ 1 yes

❑ 2 no

❑ 8 doesn’t know/doesn’t remember

D13 *(Has a doctor ever told you that you have)* asthma?

❑ 1 yes

❑ 2 no *Go to D15*

❑ 8 doesn’t know/doesn’t remember

D14 Have you ever taken prescription drugs for asthma in the past two years?

❑ 1 yes

❑ 2 no

❑ 8 doesn’t know/doesn’t remember

D15 *(Has a doctor ever told you that you have)* chronic bronchitis, emphysema or chronic obstructive pulmonary disease?

❑ 1 yes

❑ 2 no

❑ 8 doesn’t know/doesn’t remember

D16 *(Has a doctor ever told you that you have)* rheumatism, arthritis or arthrosis?

❑ 1 yes

❑ 2 no

❑ 8 doesn’t know/doesn’t remember

D17 Do you suffer from back pain that limits your daily activities or requires regular treatment?

❑ 1 yes

❑ 2 no

❑ 8 doesn’t know/doesn’t remember

D18 Have you ever had a cerebral vascular accident such as a thrombosis or a stroke?

❑ 1 yes

❑ 2 no

❑ 8 doesn’t know/doesn’t remember

D19 During the past two years, did you see a doctor for a mental health problem (depression or anxiety, for example)?

❑ 1 yes

❑ 2 no *Go to D21*

❑ 8 doesn’t know/doesn’t remember *Go to D21*

D20 Could you tell us what the exact problem was? (*If more than one, ask about the most important for the respondent*)

❑ 1 depression

❑ 2 burn-out

❑ 3 bipolar or manic-depressive disorder

❑ 4 anxiety or phobia

❑ 97 other, *specify*

❑ 98 doesn’t know/doesn’t remember

D21 During the past two years, did you see a doctor for severe digestive problems?

❑ 1 yes

❑ 2 no *Go to D23*

❑ 8 doesn’t know/doesn’t remember *Go to D23*

D22 Could you tell us what the exact problem was? (*If more than one, ask about the most important for the respondent*)

❑ 1 stomach or duodenal ulcer

❑ 2 ulcerative colitis or Crohn’s disease

❑ 3 irritable bowel

❑ 97 other, *specify*

❑ 98 doesn’t know/doesn’t remember

D23 Are you currently being followed for cancer?

❑ 1 yes

❑ 2 no

❑ 8 doesn’t know/doesn’t remember

D25 In addition to the health problems previously mentioned, do you have other health problems for which you are being followed or treated regularly?

❑ 1 yes

❑ 2 no *Go to E1*

❑ 8 doesn’t know/doesn’t remember *Go to E1*

D26 Could you tell us what it is?

**Section E – Clinical preventive services**

***Note : This section concerns only users of health services who have an eligible usual source of care (A5 or A5b = 1 or 2 or 3) located in Montréal or Montérégie.***

The following section looks at preventive health services you obtained at the («Name of the source of care»), either from a doctor or from a nurse.

**Nutrition counselling**

E1 When was the last time someone talked with you about your diet and eating habits?

❑ 1 less than 1 year ago

❑ 2 1 to 2 years ago

❑ 3 more than 2 years ago

❑ 4 it never happened *Go to E3*

❑ 8 doesn’t know/doesn’t remember *Go to E3*

E2 Were you…?

❑ 1 … asked about them without receiving any particular recommendations

❑ 2 … given recommendations on how to improve your habits

❑ 3 … or advised to keep your habits

❑ 8 doesn’t know/doesn’t remember

**Physical activity counselling**

E3 When was the last time someone talked with you about your physical activity?

❑ 1 less than 1 year ago

❑ 2 1 to 2 years ago

❑ 3 more than 2 years ago

❑ 4 it never happened *Go to E5*

❑ 8 doesn’t know/doesn’t remember *Go to E5*

E4 Were you…?

❑ 1 … asked about it without receiving any particular recommendations

❑ 2 … given recommendations on how to improve your level of activity

❑ 3 … or advised to keep it up

❑ 4 other

❑ 8 doesn’t know/doesn’t remember

**Documentation of smoking status**

Now we’re going to talk about advice you've been given concerning smoking.

E5 Do you smoke cigarettes, cigars or a pipe, every day or once in a while?

❑ 1 yes *Go to E8*

❑ 2 no

E6 Have you ever smoked?

❑ 1 yes

❑ 2 no *Go to E8*

E7 When did you quit smoking?

❑ 1 less than 1 year ago

❑ 2 between 1 and 2 years ago

❑ 3 more than 2 years ago

❑ 8 doesn’t know/doesn’t remember

**Screening for smoking status**

E8 At the («Name of the source of care»), when was the last time you were asked whether you smoked?

❑ 1 less than 1 year ago

❑ 2 between 1 and 2 years ago

❑ 3 more than 2 years ago

❑ 4 it never happened

❑ 8 doesn’t know/doesn’t remember

*Note: The following questions are for smokers or ex-smokers who quit less than 2 years ago (E5 = 1 or E7 = 1 or 2). For other respondents, go to E10*

**Smoking cessation counselling**

E9 When was the last time you were recommended to quit smoking?

❑ 1 less than 1 year ago

❑ 2 between 1 and 2 years ago

❑ 3 more than 2 years ago

❑ 4 it never happened

❑ 8 doesn’t know/doesn’t remember

**HBP screening**

The next questions are about screening for some health problems.

E10 At the («Name of the source of care»), when was the last time someone measured your blood pressure?

❑ 1 less than 1 year ago

❑ 2 between 1 and 2 years ago

❑ 3 more than 2 years ago

❑ 4 never

❑ 8 doesn’t know/doesn’t remember

**Cervical cancer screening (for women aged 18 to 69 only)**

E11 When was your last PAP test done? (*Only on demand* : *A PAP test is a test performed by a doctor or nurse; he or she examines the cervix, takes a sample using a little stick or brush, and sends the sample to the lab to screen for cervical cancer).*

❑ 1 in the last 3 years *Go to E13*

❑ 2 more than 3 years ago

❑ 3 never

❑ 8 doesn’t know/doesn’t remember

E12 Have you had a hysterectomy (that is, an operation to remove the uterus)?

❑ 1 yes

❑ 2 no

❑ 8 doesn’t know/doesn’t remember

**Breast cancer screening (for women aged 50 to 69 only)**

E13 At («Name of the source of care»), when was the last time you were advised to pass a mammography? (that is, an x-ray to detect breast cancer)

❑ 1 in the last 2 years

❑ 2 more than 2 years ago

❑ 3 never

❑ 8 doesn’t know/doesn’t remember

**Colorectal cancer screening (for people aged 50 and over only)**

The next questions are about colon and rectal cancer.

E14 When was the last time you were prescribed a test to detect blood in your stools?

❑ 1 in the last 2 years

❑ 2 more than 2 years ago

❑ 3 never

❑ 8 doesn’t know/doesn’t remember

E15 When was the last time you were advised to pass a colonoscopy? (that is, an exam where a long tube is introduced into the rectum in order to see the interior of the intestine) (If the respondent says he (she) has passed a sigmoidoscopy, consider it as a colonoscopy)

❑ 1 in the last 5 years

❑ 2 between 6 and 10 years ago

❑ 3 more than 10 years ago

❑ 4 never

❑ 8 doesn’t know/doesn’t remember

**Dyslipidemia screening (for women aged 50 to 70 or men aged 40 to 70 only)**

E16 When was the last time you were prescribed a blood test to measure your cholesterol level?

*(Only if necessary : lipids or fat in the blood)*

❑ 1 in the last 3 years

❑ 2 more than 3 years ago

❑ 3 never

❑ 8 doesn’t know/doesn’t remember

**Diabetes screening (for people aged 40 and over only)**

E17 When was the last time you were prescribed a test to measure your blood sugar level (glycaemia)?

❑ 1 in the last 3 years

❑ 2 more than 3 years ago

❑ 3 never

❑ 8 doesn’t know/doesn’t remember

**Influenza vaccination**

E18 Over the past year, were you vaccinated against influenza A H1N1 (pandemic)? (At the clinic or elsewhere)

❑ 1 yes

❑ 2 no

❑ 8 doesn’t know/doesn’t remember

E19 At the («Name of the source of care») when was the last time you were vaccinated against seasonal flu (this vaccine is usually given in the fall) or the last time you were advised to get this vaccine?

❑ 1 less than 1 year ago

❑ 2 between 1 to 2 years ago

❑ 3 more than 2 years ago

❑ 4 never

❑ 8 doesn’t know/doesn’t remember

**Section F – Sociodemographic characteristics**

Now I have a few questions that will serve to classify your answers.

F1 Were you born...?

❑ 1 in Canada

❑ 2 outside of Canada

❑ 8 doesn’t know/doesn’t remember

F2 Was one of your parents born outside Canada?

❑ 1 yes

❑ 2 no

❑ 8 doesn’t know/doesn’t remember

***Note****: The following question (F3) is only for people born outside Canada (F1 = 2)*

F3 For how many years have you been living in Canada?

❑ 0 less than one year

❑ 98 doesn’t know/doesn’t remember

F4 What language do you speak the most often at home?

❑ 1 French

❑ 2 English

❑ 3 other

F5 What is the highest level of education that you have completed or diploma you have obtained?

(*Read choices only if necessary)*

❑ 1 no diploma (elementary school)

❑ 2 high school diploma

❑ 3 diploma or certificate from a trade school or vocational school

❑ 4 diploma from a business college

❑ 5 CEGEP diploma

❑ 6 Bachelor’s degree

❑ 7 Master’s degree or PhD

❑ 97 other, *Specify*

❑ 98 doesn’t know/doesn’t remember

F6 Which statement best describes your principal occupation over the past 6 months? Is it…

❑ 1 full-time worker

❑ 2 part-time worker

❑ 3 student

❑ 4 retired

❑ 5 stayed at home

❑ 6 temporarily off work

❑ 7 employment insurance recipient (unemployed)

❑ 8 social assistance recipient

❑ 97 other, *Specify*

❑ 98 doesn’t know/doesn’t remember

F7 For statistical purpose, is the total (gross) income of your household… *(Note : household excludes roomates)*

❑ 1 …less than $15,000

❑ 2 …from 15 to $25,000

❑ 3 …from 25 to $35,000

❑ 4 …from 35 to $55,000

❑ 5 …from 55 to $75,000

❑ 6 …from 75 to $100,000

❑ 7 …$100,000 and more

❑ 98 doesn’t know/doesn’t remember

❑ 99 refused

F8 Thinking of your economic situation compared with other people your age, would you…

❑ 1 … consider that you are well off

❑ 2 … consider that your income are sufficient

❑ 3 … consider yourself poor

❑ 4 … consider yourself very poor

❑ 8 doesn’t know/doesn’t remember

❑ 9 refused

F9 Do you or does somebody in your household own a car? (long-term lease included) *(Note : household excludes roomates)*

❑ 1 yes

❑ 2 no

❑ 9 refused

F10 Do you or does somebody in your household own a house or an apartment? *(Note : household excludes roomates)*

❑ 1 yes

❑ 2 no

❑ 8 doesn’t know/doesn’t remember

❑ 9 refused

F11 Do you or does somebody in your household have an RRSP (or RRIF) or retirement savings plan? *(Note : household excludes roomates)*

❑ 1 yes

❑ 2 no

❑ 8 doesn’t know/doesn’t remember

❑ 9 refused

F12 How many people less than 18 years old usually live in your household? *(Note : household excludes roomates*)

❑ 99 refused

F12b How many people 18 years old or over, including you, live in your household? *(Note : household excludes roomates*)

❑ 99 refused

F13 Do you have private health insurance, other than the public system, that reimburses totally or partially the following items…

…your medications?

❑ 1 yes

❑ 2 no

❑ 8 doesn’t know/doesn’t remember

❑ 9 refused

F14 … dental care?

❑ 1 yes

❑ 2 no

❑ 8 doesn’t know/doesn’t remember

❑ 9 refused

F15 …complementary health services (such as certain diagnostic tests, psychological services, chiropractors, etc.)?

❑ 1 yes

❑ 2 no

❑ 8 doesn’t know/doesn’t remember

❑ 9 refused

**PRINCIPAL INVESTIGATORS**

Jean-Frédéric Levesque

Raynald Pineault

Pierre Tousignant

**COLLABORATION**

Team of researchers and partners associated with this research project

**INSTUTIONAL SUPPORT**

(1) Canadian Institutes of Health Research (CIHR); (2) Fonds de recherche en santé du Québec (FRSQ); (3) Ministère de la Santé et des Services sociaux du Québec; (4) Agence de la santé et des services sociaux de Montréal – Direction de santé publique; (5) Agence de la santé et des services sociaux de la Montérégie; (6) Institut national de santé publique du Québec (INSPQ).

This project has been approved by the research ethics committee at the Agence de la santé et des services sociaux de Montréal.

Use of this questionnaire in whole or in part is not authorized without the consent of the study investigators.
